# Stream metabolism controls diel patterns and evasion of CO_2_ in Arctic streams

**DOI:** 10.1111/gcb.14895

**Published:** 2019-11-29

**Authors:** Gerard Rocher‐Ros, Ryan A. Sponseller, Ann‐Kristin Bergström, Maria Myrstener, Reiner Giesler

**Affiliations:** ^1^ Climate Impacts Research Centre Department of Ecology and Environmental Science Umeå University Abisko Sweden

**Keywords:** Arctic, carbon cycle, carbon processing, CO_2_ evasion, stream metabolism

## Abstract

Streams play an important role in the global carbon (C) cycle, accounting for a large portion of CO_2_ evaded from inland waters despite their small areal coverage. However, the relative importance of different terrestrial and aquatic processes driving CO_2_ production and evasion from streams remains poorly understood. In this study, we measured O_2_ and CO_2_ continuously in streams draining tundra‐dominated catchments in northern Sweden, during the summers of 2015 and 2016. From this, we estimated daily metabolic rates and CO_2_ evasion simultaneously and thus provide insight into the role of stream metabolism as a driver of C dynamics in Arctic streams. Our results show that aquatic biological processes regulate CO_2_ concentrations and evasion at multiple timescales. Photosynthesis caused CO_2_ concentrations to decrease by as much as 900 ppm during the day, with the magnitude of this diel variation being strongest at the low‐turbulence streams. Diel patterns in CO_2_ concentrations in turn influenced evasion, with up to 45% higher rates at night. Throughout the summer, CO_2_ evasion was sustained by aquatic ecosystem respiration, which was one order of magnitude higher than gross primary production. Furthermore, in most cases, the contribution of stream respiration exceeded CO_2_ evasion, suggesting that some stream reaches serve as net sources of CO_2_, thus creating longitudinal heterogeneity in C production and loss within this stream network. Overall, our results provide the first link between stream metabolism and CO_2_ evasion in the Arctic and demonstrate that stream metabolic processes are key drivers of the transformation and fate of terrestrial organic matter exported from these landscapes.

## Graphical abstract

Streams play an important role in the global carbon cycle, accounting for a large portion of CO_2_ evaded from inland waters. Our results show that aquatic biological processes in Arctic streams regulate CO_2_ concentrations and evasion at multiple timescales. Photosynthesis caused CO_2_ concentrations to decrease by as much as 900 ppm during the day, and CO_2_ evasion was sustained by aquatic ecosystem respiration. This study provides the first link between stream metabolism and CO_2_ evasion in the Arctic and demonstrates that stream metabolic processes are key drivers of the transformation and fate of terrestrial organic matter exported from these landscapes.

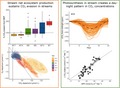

## 1 INTRODUCTION

Streams receive large amounts of carbon (C) from terrestrial ecosystems (Drake, Raymond, & Spencer, 2017) and emit a large fraction of this as CO_2_ to the atmosphere (Raymond et al., 2013). The magnitude of CO_2_ evasion from running waters is similar to the net ocean CO_2_ exchange, and therefore represents a critical component in the global C cycle (IPCC, 2013). While terrestrial organic carbon (OC) is a major source of stream CO_2_, it can be mineralized in soils and subsequently transported to streams in gas form (Öquist et al., 2009), respired within the stream ecosystem (Fisher & Likens, 1973; Hedin, 1990), and/or photo‐oxidized in the water column (Cory, Ward, Crump, & Kling, 2014). Resolving these different pathways is necessary to determine the fate of OC at regional scales, including the magnitude of CO_2_ evasion and water‐borne C export to recipient systems (Webb, Santos, Maher, & Finlay, 2018).

While processes delivering CO_2_ to streams have been extensively researched, individual studies often reach different conclusions in terms of assigning relative contribution to any one mechanism. A potential reason for these differences is that each pathway operates within distinct compartments of fluvial ecosystems, and thus, studies on specific mechanisms often fail to capture others (but see Demars, 2018; Lupon et al., 2019; Rasilo, Hutchins, Ruiz‐González, & del Giorgio, 2016). For instance, studies of photo‐oxidation suggest this as an important CO_2_ source in streams (>70%; Cory et al., 2014), but these typically consider only processes that occur in the water column. Other studies indicate that the contribution from soil respiration to stream CO_2_ evasion is more than 90% (Winterdahl et al., 2016), but these often neglect the potential role of benthic and hyporheic processes. Finally, while there has been decades of research on stream metabolism (Hoellein, Bruesewitz, & Richardson, 2013), these rates have not been integrated with estimates of CO_2_ evasion until recently (Hotchkiss et al., 2015). Overall, while the different conclusions drawn from these studies likely reveal real variation in contributing processes among systems, the large variability also reflects the challenge of partitioning these sources at meaningful spatial and temporal scales.

One way to partition the different pathways contributing to CO_2_ evasion is to couple continuous measurements of stream CO_2_ dynamics with independent and simultaneous estimates of aquatic ecosystem metabolism based on O_2_ measurements. Ecosystem metabolism in streams has been measured and modelled for decades using diel measurements of O_2_ concentrations in water (Hall & Hotchkiss, 2017; Odum, 1956). This approach assumes that the concentration of O_2_ in water is affected by three processes: (a) gross primary production (GPP) that produces O_2_, (b) ecosystem respiration (ER) that consumes O_2_ and (c) stream water turbulence that affects the air–water exchange of O_2_. Recent advances in O_2_ sensor technology, together with new modelling tools, make it possible to estimate daily GPP, ER and net ecosystem production (NEP; NEP = GPP − ER) using continuous time series of O_2_, light and hydrological parameters (Appling, Hall, Yackulic, & Arroita, 2018; Hall & Hotchkiss, 2017; Holtgrieve, Schindler, Branch, & A'mar, 2010). Importantly, GPP and ER also consume and produce CO_2_, respectively, and thus provide estimates of aquatic C processing rates that can be compared to independent measures of CO_2_. In this way, estimating metabolism modelled from O_2_ data is a powerful tool to understand CO_2_ sources to streams (Hotchkiss et al., 2015), yet few studies have coupled high frequency measurements of O_2_ and CO_2_ with the goal of resolving these different pathways (but see Gómez‐Gener, von Schiller, et al., 2016; Stets et al., 2017).

The few existing studies that address how stream metabolism may contribute to CO_2_ evasion are from boreal (Crawford, Striegl, Wickland, Dornblaser, & Stanley, 2013; Rasilo et al., 2016) or temperate ecosystems (Cole & Caraco, 2001; Crawford et al., 2014; Gómez‐Gener, von Schiller, et al., 2016; Hotchkiss et al., 2015), while studies of these processes in the Arctic tundra are lacking. Tundra streams are characterized by cold temperatures, long days with high incident light during a short summer and winters that span for more than 6 months. Yet, these streams often drain soils with large C stocks (Schuur et al., 2015), and export vast quantities of OC to the Arctic ocean (Cooper et al., 2008). Furthermore, streams represent considerable sources of CO_2_ evasion in the Arctic landscape (Lundin et al., 2016; Stackpoole et al., 2017) and emit more C than is exported to the ocean (Serikova et al., 2018). Given that climate change is drastically altering the hydrology and biogeochemistry of Arctic landscapes (Drake et al., 2018; Kendrick et al., 2018), understanding how C is mineralized and evaded within streams (e.g. Giesler et al., 2013) is necessary to understand and predict the effects of environmental change on C cycling in this region.

In this study, we ask: how do stream metabolic processes affect CO_2_ dynamics and evasion in Arctic stream networks? To answer this, we measured CO_2_ and O_2_ concentrations continuously in six streams in an Arctic catchment during the summer of 2015 and 2016. Specifically, we (a) quantified the contribution of the stream NEP to CO_2_ evasion and (b) explored whether GPP can explain diel changes in CO_2_ evasion. To achieve this, we modelled metabolic rates using the O_2_ data and estimated CO_2_ evasion simultaneously.

## 2 MATERIALS AND METHODS

### 2.1 Site description

**Figure 1:**
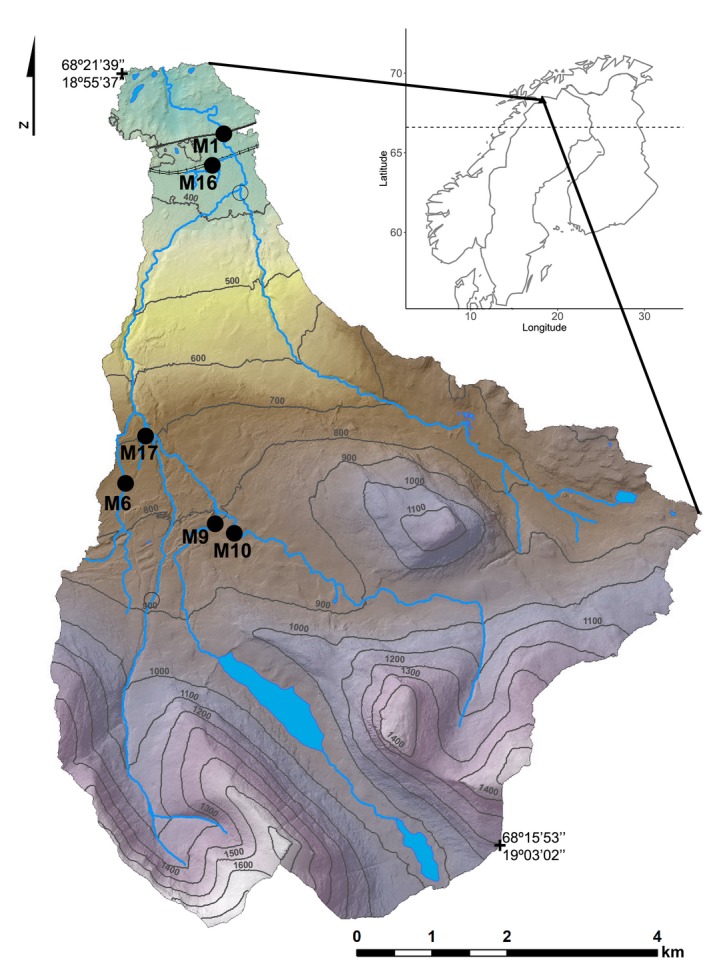
Map of the Miellajokka catchment with the coloration indicating changes in elevation. The black dots represent the location of the measuring sites in this study. The inset shows the location of the Miellajokka catchment within Scandinavia, and the dashed line represents the Arctic circle

The Miellajokka catchment (52.5 km^2^) in north‐western Sweden (Figure 1; 68°21′14″N, 18°56′16″E) is located near the Abisko Scientific Research Station. For the period 1990–2013, average annual air temperature was 0.3°C and the average annual precipitation was 337 mm (Abisko Station Meteorological Data: http://www.polar.se/abisko). Climate in the Miellajokka catchment is characterized by long winters with precipitation as snow from October to May and a short terrestrial growing season from June to September (Christensen et al., 2012). Hydrologic patterns reflect the seasonal climate regime, with a spring flood in May or June during snow melt (discharge at the outlet of 20–25 m^3^/s), and base flow of about 0.05–0.1 m^3^/s during the autumn and winter (Lyon et al., 2018). Dissolved organic carbon (DOC) in Miellajokka can reach 8–10 mg C/L during spring flood and decrease to about 2 mg C/L during summer base flow (Giesler et al., 2014). Dissolved inorganic carbon is around 4 mg C/L during summer base flow conditions, but is lower during spring flood (<2 mg C/L; Giesler et al., 2014). The pH in the catchment is circumneutral and with little seasonal variation (Giesler et al., 2013). The stream network ranges from first to fourth Strahler order streams (Table 1), with a total length of 44.6 km and a total stream surface area of 0.151 km^2^ (Rocher‐Ros, Sponseller, Lidberg, Mörth, & Giesler, 2019). Streams are moderately steep with slopes ranging from 0.07 to 0.32 m/m (Lyon et al., 2018), and several medium‐sized waterfalls. There are two lakes in the catchment, covering in total 0.69 km^2^.

**Table 1:** Physical and chemical properties of the streams monitored. Discharge, water temperature and *p*CO_2_ show the average value and the 0.05–0.95 quantile in parenthesis

Site	Elevation (m a.s.l.)	Catchment area (km^2^)	Strahler order	Discharge (L/s)	Water temperature (°C)	*p*CO_2_ (ppm)
M1	381	51.5	4	1,513 (643–3,849)	7.6 (5.2–10)	1,130 (910–1,320)
M6	747	1.8	2	101 (47–247)	6.1 (3.8–8.4)	840 (610–1,120)
M9	800	10.9	2	654 (316–1,080)	8.1 (4.1–11.9)	880 (650–1,100)
M10	815	8.6	3	361 (287–482)	7.2 (4.6–10.2)	740 (460–1,080)
M16	385	0.7	2	128 (71–208)	6.9 (5.1–8.3)	1,990 (1,700–2,230)
M17	706	0.11	1	14 (9–18)	5.4 (1.5–8.7)	2,460 (1,900–3,100)

The Miellajokka catchment is north‐facing and elevation ranges between 384 and 1,731 meters above sea level (m a.s.l.). In this region, sporadic permafrost occurs at low elevations and discontinuous to continuous permafrost at high elevation zones (Gisnås et al., 2017). At elevations above 1,200 m a.sl., the land is mostly barren, with several permanent snowfields. Between 700 and 1,200 m, the landscape is characterized by tundra vegetation and cryoturbated soils (Becher, Olid, & Klaminder, 2013). The tree line is at approximately 700 m, and below this elevation, the landscape consists of sparse mountain birch forest (*Betula pubescens* spp. *Czerepanovii*) with mixed tundra heath vegetation. Below 400 m, there is a more productive birch forest with a denser canopy cover. The sites M1 and M16 are located here, and the riparian forest cover results in less incident light compared to streams draining tundra vegetation (Myrstener et al., 2018).

### 2.2 Continuous measurements

We recorded water temperature, water level and dissolved concentrations of CO_2_ and O_2_ at six stream locations from late June to early September in 2015 and 2016 (Figure 1). Water temperature and water level were recorded hourly using HOBO water level loggers (model U20‐001‐04; Onset Computer Corporation). Stream CO_2_ concentrations were measured hourly using infrared gas analyser (IRGA) adapted for wet environments. In streams M1 and M16, we used a Vaisala GMT220 sensor (Vaisala) covered with a PTFE layer highly permeable to dissolved gasses but not to water, following (Johnson et al., 2010). At sites M6, M9, M10 and M17, we used eosGP CO_2_ concentration probes (Eosense Inc.). The eosGP sensor uses the same technique as the Vaisala, but with a PTFE membrane included by design. The Vaisala and eosGP sensors were connected to CR1000 data loggers (Campbell Scientific Inc.), powered with 12 V lead–acid batteries. The sensors were calibrated with standard gases in the lab before and after deployment in the field, using gas concentrations of 400, 2,000 and 5,000 ppm of CO_2_. Sensors were placed with protective casings to avoid damage due to floods and rock movements in the water and were inspected and gently cleaned every 3 weeks. Due to the fragile material of the membrane and the extreme conditions in some streams, several malfunctions and subsequent data loss occurred, particularly at M1. We monitored O_2_ concentrations every 10 min using miniDOT oxygen loggers (Precision Measurement Engineering Inc.). The loggers were installed with a copper mesh to avoid biofouling, and the sensor was placed in the opposite direction of the flow to prevent accumulation of debris and impact of stones. Prior and post deployment, the sensors were intercalibrated using reaerated water to achieve a 100% saturation of O_2_, and then by adding dry yeast to decrease the O_2_ saturation to 0%.

All loggers were attached in the stream using a perforated steel pipe attached to a heavy metal platform to prevent movement. The temperature/water level loggers were placed firmly inside the pipe, the CO_2_ sensor outside but downstream of the pipe, to be exposed to flowing water, and the O_2_ sensor parallel to the flow with the sensor facing downstream. We selected these sites taking into account three criteria: (a) a suitable location within the thalweg to install loggers so that they would not be exposed to air during base flow conditions, while also avoiding deep pools; (b) lack of upstream tributaries (in all streams except M16 the distance to the nearest tributary was >1 km); and (c) minimal groundwater inputs immediately upstream of deployment sites. On two to six occasions, we quantified local groundwater inputs by comparing discharge estimates made with salt slugs at the deployment site with those made upstream; 50–500 m, depending on stream size. For each site, we observed similar discharge values, differing less than 10%, for example, the precision of the slug discharge measurements (Moore, 2005). This indicates low rates of groundwater input within the likely footprint of the metabolism estimate.

Snow/ice cover and peak flow conditions during snow melt restricted the time period of our measurements to June–September. Due to these climatic constraints, in 2015, we installed the loggers between 5 and 7 July until 7 September, and in 2016, between 15 and 17 June until 8 September. Other climatic variables used in this study were atmospheric air pressure and light irradiance. We used data measured in the meteorological station in Stordalen (SITES Sweden monitoring station, circa 4 km from the catchment outlet). To obtain atmospheric pressure in each site, the atmospheric pressure was corrected by the elevation difference following the barometric formula (Hall & Hotchkiss, 2017).

### 2.3 Discharge and the gas exchange coefficient (*K*_600_)

Discharge (*Q*) was measured at every site on several occasions with the salt slug method (Moore, 2005). At M1, M6 and M16, we obtained more than 10 measurements, while in sites M9, M10 and M17, we performed four discharge measurements. The discrete measures were then related to depth that was continuously monitored with a pressure logger to obtain continuous discharge estimates. The relationship between depth and discharge used was linear, with an *R* ^2^ > .85 in all streams. To relate the depth of the logger position to the average channel depth, we measured depth every 5–20 cm (depending on the stream size) along 8–10 cross sections upstream of the sensors at each site.

The gas exchange coefficient (*K*_600_) was primarily obtained using the night‐time regression method (Hornberger & Kelly, 1975; Odum, 1956). Briefly, at sunset when GPP approaches zero, O_2_ in water decreases as there is no biological input. The rate of decrease in O_2_ concentrations is therefore dependent on the rate in which O_2_ can reach a new equilibrium with the atmosphere, and thus proportional to the *K*_600_. During the period when this occurs, $K_{O_{2}}$ is approximated by the slope of the relationship between the rate of change in O_2_ concentration and the O_2_ deficit in the water (Odum, 1956), that can be converted to *K*_600_ (Aristegi, Izagirre, & Elosegi, 2009). Given that the length of the night shifts strongly through the summer at high latitudes, we used an algorithm to perform six linear regressions each day at different periods to capture the night‐time drop of O_2_ using an R script (https://github.com/rocher-ros/nighttime_regression_multiple). We selected days when night was at least 2 hr long (from 25 July onwards), and days when the night‐time regression had an *R* ^2^ > .7. The *K*_600_ values obtained with this method were then related to discharge, usually a major predictor of *K*_600_ within a site (Raymond et al., 2012). In our case, *K*_600_ was significantly related to discharge in all sites (Figure S4). At three sites, we also performed several propane releases, following the method described in Wallin et al. (2011), and the *K*_600_ obtained by this method agreed well with the night‐time regression estimates (Figure S4a,b,e).

To calculate the specific gas aeration coefficients for O_2_ and CO_2_, $K_{O_{2}}$ and $K_{{\text{CO}}_{2}}$, we used the following approach. The *K*_600_ is a standardized measure of the gas exchange coefficient for a Schmidt number of 600, which can be converted to a specific gas following (Wanninkhof, 1992):

(1) $$K_{x}=K_{600}/{\left(600/{\text{SC}}_{x}\right)}^{-0.5},$$

where *K_x_* is the gas exchange coefficient for a given gas *x*, and SC*_x_* is the Schmidt number of that gas (in this study CO_2_ or O_2_). The Schmidt numbers for each gas were calculated using the published Schmidt coefficients (Raymond et al., 2012), for O_2_ was calculated as:

(2) $${\text{SC}}_{O_{2}}=1800.6-\left(120.1\times T\right)+\left(3.78\times T^{2}\right)-\left(0.0476\times T^{3}\right).$$

And for CO_2_ as:

(3) $${\text{SC}}_{{\text{CO}}_{2}}=1911.1-\left(118.1\times T\right)+\left(3.45\times T^{2}\right)-\left(0.0413\times T^{3}\right),$$

where *T* is the water temperature in °C. With the gas specific Schmidt numbers (Equations 2 and 3), it was therefore possible to calculate the $K_{O_{2}}$ and the $K_{{\text{CO}}_{2}}$ (Equation 1).

### 2.4 Stream metabolism modelling

Stream metabolism was modelled based on the open channel diel oxygen method (Odum, 1956). Stream NEP is the balance between GPP and ER, and these two processes affect the diel oxygen concentration. These diel patterns can be used to estimate GPP and ER by analysing O_2_ time series. We used a Bayesian inverse model from Hall and Hotchkiss (2017), governed by the following equation:

(4) $$O_{2_{t}}=O_{2_{t-1}}+\left(\frac{\text{GPP}}{z}\times \frac{{\text{PAR}}_{t-1}}{\sum_{t=0}^{t=24}\text{PAR}}\right)+\frac{\text{ER}\times \Delta t}{z}+K_{O_{2}}\times \left(O_{2_{\text{sat}\left(t-1\right)}}-O_{2_{\left(t-1\right)}}\right)\times \Delta t,$$

where $O_{2_{t}}$ is the oxygen concentration at time *t* (in g O_2_/m^3^), *z* is the channel depth (in m), PAR is the photosynthetically active radiation (in mol m^−2^ s^−1^), $K_{O_{2}}$ is the gas exchange coefficient of O_2_ (in day^−1^), Δ*t* is the time steps of the time series (10 min) and $O_{2_{\text{sat}}}$ is the concentration of O_2_ in the water if it would be 100% saturated. GPP and ER are obtained as areal rates (g O_2_ m^−2^ day^−1^) and were converted to C assuming that 1 mol of O_2_ is produced/consumed for 1 mol of CO_2_ (Demars et al., 2016). We acknowledge that the conversion between O_2_ and CO_2_ depends on the chosen respiratory or photosynthetic quotient and could thus bias results (Berggren, Lapierre, & Del Giorgio, 2012; Williams & Robertson, 1991).

We modelled the three parameters (GPP, ER and $K_{O_{2}}$), but using priors for *K* that were strongly constrained to minimize the problem of equifinality (Appling et al., 2018). Models that predict the three parameters avoid errors associated with estimating *K*_600_ empirically (Aristegi et al., 2009; Holtgrieve, Schindler, & Jankowski, 2016), but can give multiple solutions where different combinations of GPP, ER and *K*_600_ reproduce the same O_2_ data, so‐called equifinality (Appling et al., 2018). A solution for this is to relate *K*_600_ to hydrological measures such as discharge, which should be a proxy for *K*_600_ within a site (Appling et al., 2018). We used the relationship between *K*_600_ and *Q* from each site obtained from the night‐time regression method (see above), to obtain an approximate *K*_600_ for each day with its error associated. Then, for each day, the prior distribution of $K_{O_{2}}$ was defined by the mean and standard deviation (*SD*) obtained from the *K*_600_–Q relationship (Figure S4). The priors for GPP and ER were largely uninformed, with a mean of 1 and −5 g O_2_ m^−2^ day^−1^, respectively, and an *SD* of 2. The priors of GPP and ER were chosen to be similar to the mean values measured in another Arctic stream in Alaska (Huryn, Benstead, & Parker, 2014). To simulate the posterior distributions of the parameters, we used the *metrop()* function of the *mcmc* package in R (R Core Team, 2017; version 3.4). Each model was run 150,000 times for each day and used the last 100,000 simulations to assure the convergence of the posterior distributions, based on visual observations. All metabolism computations were performed following Hall and Hotchkiss (2017), using a modified version of the R script available in that publication.

We further filtered the modelled estimates of metabolism through the following quality tests: (a) we calculated the mean average error (MAE) between the observed and the modelled O_2_ concentrations. If the MAE was larger than 0.2, we discarded that day. The threshold of 0.2 was determined after visually inspecting the plot of O_2_ concentrations and was similar to the threshold used in another study (Lupon et al., 2019). (b) One of the model assumptions is that depth and *K*_600_ are constant throughout the day (Odum, 1956). We removed days when depth (which is also the proxy used for *K*_600_) changed more than 10% within the day. (c) Finally, daily outputs were plotted to visually inspect that the model reproduced O_2_ concentrations accurately. Here, we inspected each day manually and removed any days showing poor model fit. After this, 165 observations of daily metabolism were removed from a total of 875.

### 2.5 Estimating CO_2_ evasion

The CO_2_ exchange with the atmosphere ($E_{{\text{CO}}_{2}}$) was calculated as (Raymond et al., 2012):

(5) $$E_{{\text{CO}}_{2}}=K_{{\text{CO}}_{2}}\times z\times \left({\left[{\text{CO}}_{2}\right]}_{w}-{\left[{\text{CO}}_{2}\right]}_{a}\right),$$

where $K_{{\text{CO}}_{2}}$ (in day^−1^) is the gas exchange coefficient, *z* is the channel depth (in m), [CO_2_]_w_ is the concentration of CO_2_ measured in the water and [CO_2_]_a_ is the CO_2_ concentration in equilibrium with the atmosphere (in mol/m^3^). We used an atmospheric CO_2_ concentration of 380 ppm, obtained from the mean of several air CO_2_ measurements performed in the field. The concentrations of CO_2_ in mol/m^3^ were calculated using the *p*CO_2_ measurements and Henry's law, using the temperature measured in the oxygen sensor. The units of $E_{{\text{CO}}_{2}}$ were converted from mol C m^−2^ day^−1^ to g C m^−2^ day^−1^.

### 2.6 Mass balance along a single stream reach

In a previous study in this catchment, we measured CO_2_ evasion and discharge at a high spatial resolution (Rocher‐Ros et al., 2019). Here, we used this data set to do mass balance calculations for CO_2_ in order to generate estimates of net CO_2_ production along a stream reach (2.1 km) that loses a major fraction of water into the nearby forest as it crosses an alluvial deposit (Figure S13). We used these independent estimates and compared them with estimates derived from metabolism modelling. Along this reach, CO_2_ concentrations, discharge, *K*_600_ and channel hydraulics (wetted width, depth and velocity) were measured every 300–480 m. Therefore, it is possible to use a mass balance calculation for CO_2_ within each segment of this reach:

(6) $$C_{\text{out}}=C_{\text{in}}+C_{\text{GW}}+P-E,$$

where *C*_out_ is the CO_2_ leaving the segment, calculated as the product of discharge at the downstream end (*Q*_out_; in m^3^/day) and the CO_2_ concentration (${\text{CO}}_{2_{\text{out}}}$; in g C/m^3^); *C*_in_ is the CO_2_ entering the segment, calculated as the product of discharge at the entrance (*Q*_in_; in m^3^/day) and the CO_2_ concentration (${\text{CO}}_{2_{\text{in}}}$; in g C/m^3^); *C*_GW_ is the CO_2_ input from groundwater (GW), as the product of groundwater flow (*Q*_GW_) and the groundwater CO_2_ concentration (${\text{CO}}_{2_{\text{GW}}}$; in g C/m^3^); *P* is the production of CO_2_ within the stream segment; and *E* is evasion of CO_2_ in the stream segment. Thus, to estimate the unknown *P* (stream production of CO_2_), Equation (6) can be rearranged as:

(7) $$P=C_{\text{out}}-C_{\text{in}}-C_{\text{GW}}+E.$$

Equation (7) can be further decomposed in its components as:

(8) $$P=Q_{\text{out}}\times {\text{CO}}_{2_{\text{out}}}-Q_{\text{in}}\times {\text{CO}}_{2_{\text{in}}}-Q_{\text{GW}}\times {\text{CO}}_{2_{\text{GW}}}+E_{{\text{CO}}_{2}}\times A,$$

where $E_{{\text{CO}}_{2}}$ is the CO_2_ evasion rate (in g C/m^2^) using the average CO_2_ concentration and *A* is the stream segment area (in m^2^). *Q*_GW_ can be estimated as the difference between *Q*_in_ and *Q*_out_ for each stream segment. Importantly, because this is a losing reach, there is no net increase in groundwater contribution; therefore, all CO_2_ produced originated within the stream channel, and the parameter ${\text{CO}}_{2_{\text{GW}}}$ is the mean stream CO_2_ concentration. All these parameters were measured in the field and therefore used to estimate the internal stream CO_2_ production (*P*).

### 2.7 Data analysis and statistics

All data were analysed using R (R Core Team, 2017; version 3.5.1), the data set with daily summary data and an R script to reproduce these figures can be found in the Supporting Information. Linear regressions were performed to test the prediction that GPP is related to diel changes in CO_2_ evasion. The ΔCO_2_ to summarize the diel change in CO_2_ concentration was calculated as the difference between the highest and lowest CO_2_ concentration within each day. The diel change in CO_2_ evasion was calculated as the cumulative CO_2_ evasion occurring between sunrise and sunset, and subtracting the CO_2_ evasion before sunrise. The coefficient of variation (CV) was calculated as *SD*/average × 100, where *SD* is the standard deviation. Significant differences refer to the *p* < .05 level unless otherwise stated.

## 3 RESULTS

### 3.1 Physical and chemical characteristics of streams

**Figure 2:**
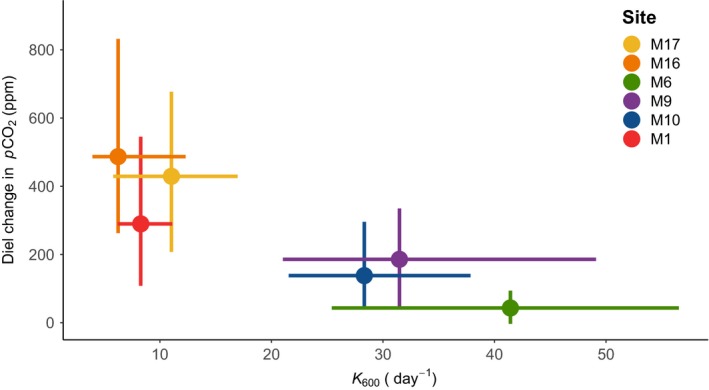
The diel change in CO_2_ concentration (ΔCO_2_) for each site in relationship to *K*_600_. Each point represents the average of each site, while the bars denote the 0.05–0.95 quantiles for both ΔCO_2_ and *K*_600_

**Figure 3:**
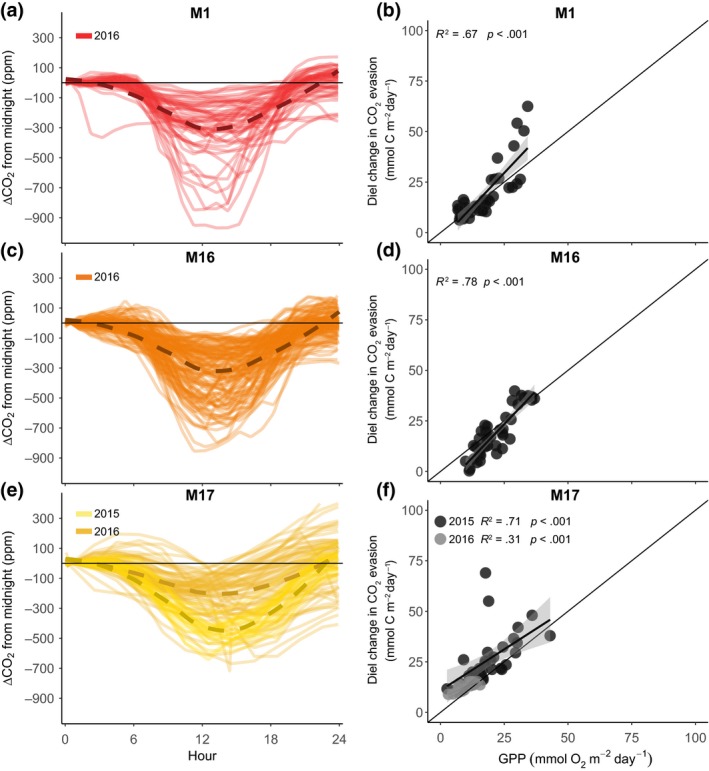
Daily variations in *p*CO_2_ (panels a, c and e) and the relationship between the diel change in CO_2_ evasion and gross primary production (GPP; panels b, d and f), in the three streams with low *K*_600_ (Figure 2). ΔCO_2_ is the daily change in *p*CO_2_ from midnight, where each solid line represents 1 day, and the dashed lines denote the average for years 2015 and 2016. The solid lines in panels (b), (d) and (f) are the linear regressions between the diel change in CO_2_ evasion and GPP

**Figure 4:**
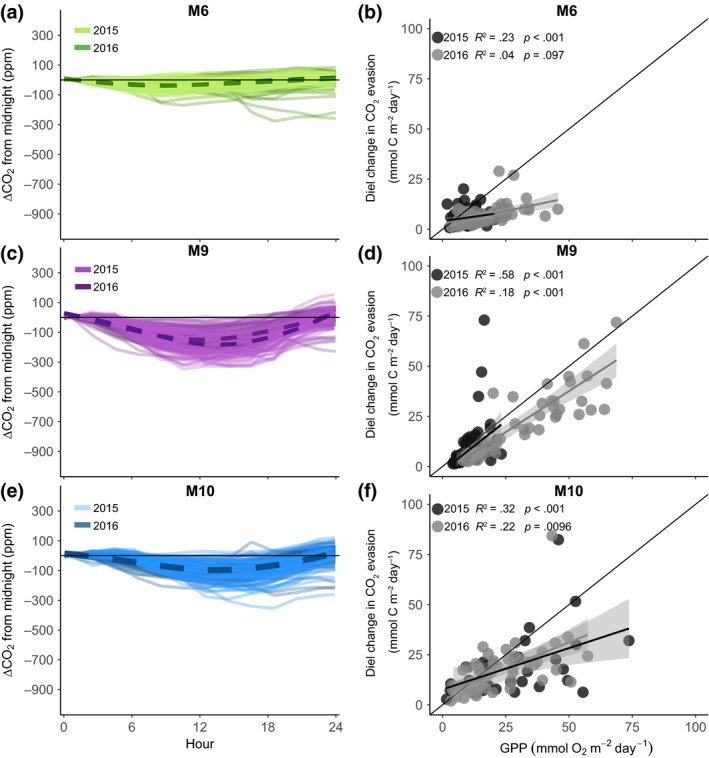
Daily variations in *p*CO_2_ (panels a, c and e) and the relationship between the diel change in CO_2_ evasion and gross primary production (GPP; panels b, d and f), in the three streams with high *K*_600_ (Figure 2). The ΔCO_2_ is the daily change in *p*CO_2_ from midnight, where each solid line represents 1 day, and the dashed lines denote the average for years 2015 and 2016. The solid lines in panels (b), (d) and (f) are the linear regressions between the diel change in CO_2_ evasion and GPP

Overall, streams were clearly separated by *K*_600_, with the more turbulent sites (M6, M9 and M10) having the highest values, ranging between 21 and 57 day^−1^ (Figure 2). By contrast, in less turbulent streams (M1, M16 and M17), *K*_600_ values were considerably lower, that is, ranging between 4 and 17 day^−1^ (Figure 2). Henceforth, the streams are labelled so that those that have a low *K*_600_ (M1_LK_, M16_LK_, M17_LK_) are easily separated for those with high *K*_600_ (M6_HK_, M9_HK_, M10_HK_). All streams were supersaturated in CO_2_, with average concentrations ranging from 740 to 2,460 ppm (Table 1). We observed the highest average CO_2_ concentrations in the two smallest streams, M17_LK_ and M16_LK_, and the lowest concentration in M10_HK_ (Table 1). The amplitude of diel change in CO_2_ concentration ranged from 0 to 920 ppm; this varied throughout the measuring periods, but also differed markedly among streams (Figure S1). Specifically, the diel change in *p*CO_2_ (ΔCO_2_) was more pronounced in M1_LK_, M16_LK_ and M17_LK_ compared to M6_HK_, M9_HK_ and M10_HK_ (Figures 3 and 4). The average diel change of *p*CO_2_ in M1_LK_, M16_LK_ and M17_LK_ was 290, 490 and 430, respectively (Figure 3a,c,e), with changes as large as 920 and 870 ppm in M1_LK_ and M16_LK_ respectively.

### 3.2 Stream metabolic rates

Rates of ER were an order of magnitude higher than GPP (Figure S6). Average ER across all streams was −1.8 g C m^−2^ day^−1^, with individual site averages ranging from −1.35 (M17_LK_) to −2.63 g C m^−2^ day^−1^ (M1_LK_, Table 2). Temporal variation in ER, described by the % CV, was greatest at M10_HK_ (59%) and lowest at M1_LK_ (27%). Average GPP across all streams was 0.22 g C m^−2^ day^−1^ with averages for individual sites ranging from 0.19 to 0.28 g C m^−2^ day^−1^ (Table 2). GPP also varied over time within sites with the highest % CV in M9_HK_ (85%) and the lowest in M16_LK_ (41%). GPP and ER were significantly and linearly related in four of the six sites, with a degree of explanation (*R* ^2^) ranging from .28 to .5 (Figure S9). In all sites, we found that ER was linearly related with discharge, with an *R* ^2^ ranging from .65 to .93 (Figure S10). GPP was also significantly related to discharge in four of the six sites, with an *R* ^2^ ranging from .22 to .64 (Figure S11). There was also a strong relationship between ER with *K*_600_ in all sites, with an *R* ^2^ ranging from .62 to .87 (Figure S12).

**Table 2:** Average values for net ecosystem production (NEP), gross primary production (GPP), ecosystem respiration (ER) and CO_2_ evasion rates of the streams. The 0.05–0.95 quantile in parenthesis. The last column indicates the mean CO_2_ evasion at day and night (noon/midnight)

Site	*N* (days)	NEP (g C m^−2^ day^−1^)	GPP (g C m^−2^ day^−1^)	ER (g C m^−2^ day^−1^)	CO_2_ evasion (g C m^−2^ day^−1^)	Noon/midnight CO_2_ evasion (g C m^−2^ day^−1^)
M1	39	−2.4 (−1.8 to −3.7)	0.21 (0.09–0.38)	−2.63 (−1.9 to −3.9)	1.3 (1.0–1.9)	1.02/1.51
M6	132	−1.9 (−0.8 to −3.6)	0.15 (0.04–0.33)	−2.08 (−0.9 to −3.9)	1.5 (0.8–2.2)	1.53/1.55
M9	135	−1.4 (−0.5 to −3.5)	0.24 (0.07–0.69)	−1.29 (−0.5 to −2.6)	1.3 (0.4–3.2)	1.21/1.52
M10	88	−1.3 (−0.4 to −2.7)	0.28 (0.05–0.62)	−1.61 (−0.6 to −3.2)	0.8 (0.3–1.4)	0.75/0.93
M16	46	−2.1 (−1.3 to −3.7)	0.24 (0.11–0.43)	−2.37 (−1.5 to −3.9)	2.7 (1.5–4.9)	2.19/3.01
M17	53	−1.2 (−0.5 to −1.9)	0.19 (0.06–0.36)	−1.35 (−0.6 to −2.2)	1.4 (0.6–2.2)	1.26/1.67

### 3.3 GPP and diel patterns of CO_2_ concentration and evasion

All streams had higher CO_2_ concentrations at night compared to day, displaying a clear diel change in *p*CO_2_ (ΔCO_2_; Figures 3 and 4). The diel pattern in *p*CO_2_ resulted in higher night‐time CO_2_ evasion rates compared to daytime rates (Table 2). Not surprisingly, this effect on CO_2_ evasion was highest in the streams with a strong diel pattern in *p*CO_2_ (Figure 3). Specifically, CO_2_ evasion at midnight compared to noon was 45%, 37% and 34% higher in sites M1_LK_, M16_LK_ and M17_LK_ respectively. The impact on CO_2_ evasion for the streams with a weaker diel *p*CO_2_ pattern was lower but still important, with midnight evasion rates 26% and 24% higher than noon in sites M9_HK_ and M10_HK_ respectively. In site M6_HK_, the *p*CO_2_ diel pattern was the weakest, and CO_2_ evasion rates at midnight were just 1% higher than noon. The magnitude of diel change in evasion was positively related to GPP rates in all streams, with significant relationships in all cases except M6_HK_ during the year 2015 (Figures 3 and 4). For the streams with large diel changes in *p*CO_2_ (M1_LK_, M16_LK_ and M17_LK_), GPP explained between 31% and 78% of the variability in the diel change in CO_2_ evasion (Figure 3b,e,f). For the streams with low *K*_600_, the degree of explanation of GPP was weaker, ranging from 4% to 58% (Figure 4b,e,f).

Furthermore, the effect of GPP was also visible directly on diel changes in *p*CO_2_ in the three streams with low *K*_600_. In M1_LK_, GPP explained 74% and in M16_LK_ 83% of the amplitude in ΔCO_2_, with values close to the 1:1 line (Figure S8). For stream M17_LK_, GPP also had a significant, linear relationship with the diel pattern in *p*CO_2_ and explained 65% and 32% of the variability for the years 2015 and 2016 respectively (Figure S8). In the other three streams, the diel change in *p*CO_2_ was lower, with an average of 40 (M6_HK_), 190 (M9_HK_) and 140 ppm (M10_HK_; Figure 4a,c,e). At these sites, where *K*_600_ was higher than 20 day^−1^, and hence, streams were more turbulent, GPP had no significant relationship with the diel change in CO_2_ concentrations (Figure S8).

### 3.4 CO_2_ evasion and the contribution of stream metabolism

**Figure 5:**
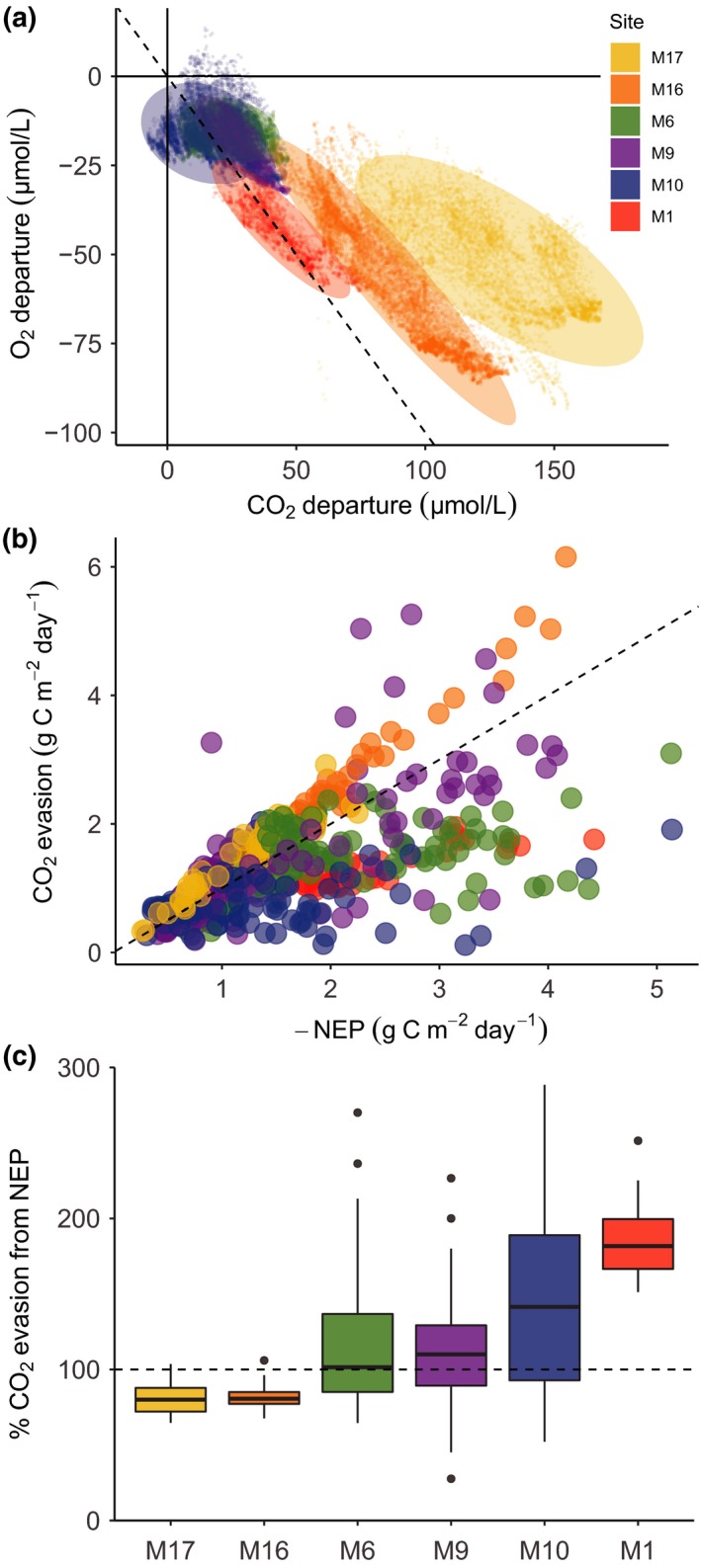
The coupling between O_2_ and CO_2_ in Arctic streams, and the contribution of net ecosystem production (NEP) to CO_2_ evasion. (a) Departure from atmospheric equilibrium of CO_2_ and O_2_, where each point is an individual hourly observation and the ellipse for each site represents the 0.95 confidence level. (b) CO_2_ evasion and NEP (with inverted sign) values for each day and all sites. Both parameters have the same units, and the dashed line is the 1:1 line where CO_2_ evasion is equal to NEP. Therefore, points below the line have higher NEP than evasion. (c) Boxplots of the proportion of CO_2_ evasion corresponding to stream NEP for each site, sorted from smallest (M17) to largest (M1) catchment area

Average daily CO_2_ evasion rates of the sites ranged from 0.1 to 6.2 g C m^−2^ day^−1^ (Table 2), with an average of 1.4 g C m^−2^ day^−1^. The highest average evasion rate was at M16_LK_ and the lowest at M10_HK_ (Table 2). All streams were undersaturated with O_2_ and supersaturated with CO_2_ relative to the atmosphere (Figure 5a). Consequently, all streams had negative NEP (ER > GPP) and were therefore net sources of CO_2_, with NEP rates comparable to CO_2_ evasion rates (Figure 5b). Average NEP among streams ranged from −1.2 to −2.4 g C m^−2^ day^−1^ (Table 2). The median contribution of NEP to CO_2_ evasion across sites varied from 80% in M17_LK_ to 182% in M1_LK_ (Figure 5c). The sites that had clear diel patterns in CO_2_ (M1_LK_, M16_LK_ and M17_LK_) were the sites that also had strong, significant linear relationships between NEP and CO_2_ evasion, with *R* ^2^ values of .74, .96 and .71 respectively (Table S1). This relationship was much weaker in the sites with a low diel pattern in CO_2_ but still significant for sites M6_HK_ and M10_HK_, with *R* ^2^ of .1 and .23, while for site M9_HK_, the relationship was not significant.

A direct comparison of departures from equilibrium of O_2_ and CO_2_ concentrations also captured similar results but without the effect of *K*_600_ and its potential uncertainties (Figure 5a). All streams were close to the 1:1 line and were significantly related, with the highest *R* ^2^ found in the streams M1_LK_, M9_HK_, M16_LK_ and M17_LK_ (.73, .5, .85 and .45, respectively), while for M6_HK_ and M10_HK_, the *R* ^2^ was .06 and .07 respectively. Although the departure from O_2_ and CO_2_ equilibrium does not incorporate the effect of the *K*_600_, its effect determines the potential for the departure. Here, the streams with high *K*_600_ (M6_HK_, M9_HK_, M10_HK_) are closer to saturation for both O_2_ and CO_2_ than the streams with low *K*_600_ (M1_LK_, M16_LK_, M17_LK_). Additionally, the spread along the 1:1 line was also larger for low compared to high *K*_600_ streams (Figure 5a). The streams M16_LK_ and M17_LK_ showed an offset relative to the 1:1 line, indicating that there is an external source of CO_2_ uncoupled from O_2_ dynamics. This external source of CO_2_ for these same streams is also detected by comparing NEP and CO_2_ evasion rates (Figure 5c), where NEP accounts for <100% of CO_2_ evasion rates.

### 3.5 CO_2_ mass balance along a stream reach

**Figure 6:**
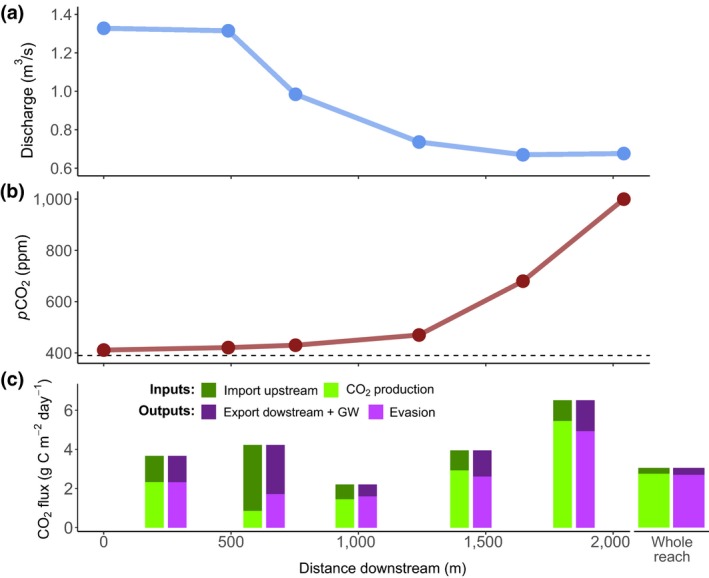
Patterns of CO_2_ concentrations and fluxes in a losing water stream. (a) Downstream change in discharge along the 2 km stream reach. The stream reach loses water as it passes through an alluvial deposit (see Figure S13 for a spatial version of this figure). We therefore expect that the contribution of terrestrially respired CO_2_ is negligible as there are no groundwater inputs. (b) How the *p*CO_2_ increases a twofold along this reach. By assuming that lateral inputs are negligible, we can do a mass balance to quantify the CO_2_ produced within the stream. (c) Calculated inputs and export of CO_2_ for five stream segments of the 2 km stream reach. The CO_2_ was produced at a rate of 2.6 g C m^−2^ day^−1^ in this reach, and the net ecosystem production the same day measured at the site M1 (~800 m downstream) was 2.8 g C m^−2^ day^−1^

Mass balance calculations along single segments of the stream reach provided evidence for net CO_2_ production within the stream. The water lost along the entire reach (Figure S13) was more than 50% (Figure 6a), as discharge decreased from 1.33 to 0.68 m^3^/s. Along this same distance, *p*CO_2_ increased more than twofold, from 400 to 1,000 ppm (Figure 6b). *K*_600_ also decreased markedly along the reach, with *K*_600_ values dropping from 54.3 to 7.8 day^−1^. CO_2_ produced within five individual segments (300–480 m) ranged from 0.86 to 5.46 g C m^−2^ day^−1^, with an average of 2.6 g C m^−2^ day^−1^ (Figure 5c). This average CO_2_ production in the reach was similar to the average CO_2_ evasion (2.7 g C m^−2^ day^−1^) and close to the NEP (−2.81 g C m^−2^ day^−1^) measured the same day at the site M1_LK_ which is 800 m downstream of this reach.

## 4 DISCUSSION

In this study, we simultaneously assessed continuous O_2_ and CO_2_ data to show that aquatic biological processes play an important role in the C cycle of these Arctic streams. In the Swedish northern landscape, the signature of aquatic metabolism was imprinted upon stream CO_2_ dynamics in two distinct ways: photosynthesis created a clear day–night difference in CO_2_ evasion and in‐stream respiration sustained CO_2_ evasion from streams throughout the summer. Streams were consistently heterotrophic, indicating that respiration in these ecosystems relies on organic C exported from land. Thus, through both autotrophic and heterotrophic processes, aquatic metabolism has the potential to regulate the transformation and the fate of terrestrial organic matter exported from Arctic landscapes.

### 4.1 Diel patterns in CO_2_ evasion

We observed a consistent and sometimes dramatic day–night change in *p*CO_2_ (Figure 3) with night‐time evasion rates that were between 24% and 45% higher than during the day in five of the six streams, a similar magnitude as reported in other studies in lower latitude regions (Peter et al., 2014; Reiman & Jun Xu, 2018; Schelker, Singer, Ulseth, Hengsberger, & Battin, 2016). Our results further indicate that this diel change in CO_2_ evasion was caused by photosynthetic activity during the day (Figures 3 and 4). The effect of GPP was also visible directly in diel changes in *p*CO_2_, but only in streams with less turbulence and lower *K*_600_ (Figure S8). This suggests that degassing in more turbulent streams conceals the effect of GPP on stream CO_2_ concentrations, as observed for O_2_ concentrations (Appling et al., 2018). Regardless, despite relatively low GPP rates (Figure S6), photosynthesis acts as important, short‐term C sink in these streams. Furthermore, this day–night pattern implies that estimates of CO_2_ evasion based on daytime observations may grossly underestimate the total daily efflux, in this study by as much as 27%. By showing how low and high *K*_600_ environments differ in their capacity to support strong diel patterns, these results may help to correct regional and global estimates of CO_2_ evasion.

Our results show that aquatic photosynthesis drives diel changes in CO_2_ evasion and *p*CO_2_ in these Arctic streams (Figures 3 and 4). However, in the Alaskan Arctic, it has been suggested that photo‐oxidation can account for as much as 70%–95% of the CO_2_ production in the water column of streams and rivers (Cory et al., 2014). If this light‐dependent process was the main driver of CO_2_ production in our streams, we would expect to see an increase in *p*CO_2_ from night to day, that is, in contrary to our observations (Figure 3). The discrepancy of our results with Cory et al. (2014) could be due to the clear, low DOC water in Miellajokka streams (Giesler et al., 2014), as compared to the more coloured and DOC rich waters in Alaska. Still, photochemical measurements are performed in the water column, which represents a minor fraction (<5%) of C mineralization from benthic and hyporheic sediments (Demars, 2018). Indeed, even considering the highest rate of photo‐oxidation from Alaska (0.3 g C m^−2^ day^−1^; Cory et al., 2014), this process would only account for 20% of average CO_2_ evasion in our streams, and an even lower fraction in other Arctic sites that have reported considerably higher evasion rates (Denfeld, Frey, Sobczak, Mann, & Holmes, 2013; Lundin, Giesler, Persson, Thompson, & Karlsson, 2013; Serikova et al., 2018).

### 4.2 Contribution of stream NEP to CO_2_ evasion

While GPP can have a strong impact on stream CO_2_ dynamics, rates of ER were an order of magnitude higher (Table 2), and therefore had a stronger overall effect on the stream C cycle. Indeed, NEP in our streams was strongly negative due to high ER rates, a common observation across riverine ecosystems (Hoellein et al., 2013), and was the major contributor to CO_2_ evasion (Figure 5). This indicates that these streams mineralize substantial amounts of the organic C received from land that otherwise would have been exported downstream to lakes or marine systems. Our reported values of the contribution of aquatic NEP to CO_2_ evasion are high compared to other studies of small streams in high latitudes (40%–75%; Lupon et al., 2019; Rasilo et al., 2016), and typically the largest contributions to date have been observed for considerably larger rivers (85%–97%; e.g. Cole & Caraco, 2001; Lynch, Beatty, Seidel, Jungst, & DeGrandpre, 2010). Therefore, our results seemingly contradict the expected minor contribution of stream NEP to CO_2_ evasion in headwaters (Hotchkiss et al., 2015), although we did find an increase in the average contribution of NEP with stream size (Figure 5c).

The discrepancy of our results with other studies reporting smaller contribution of aquatic NEP to CO_2_ evasion may reflect constraints imposed on site selection when estimating stream metabolism. Importantly, we avoided reaches for metabolism modelling that had high rates of groundwater input and/or areas with very high gas exchange (e.g. waterfalls), which are both likely hotspots of C inputs or evasion (Lupon et al., 2019; Rocher‐Ros et al., 2019). This decision may explain our relatively low CO_2_ evasion rates compared to other studies in the Arctic (Denfeld et al., 2013; Lundin et al., 2013; Serikova et al., 2018). Even within the Miellajokka catchment, the CO_2_ evasion rates observed here are lower (median 1.4 g C m^−2^ day^−1^; Table 2) than those reported in a previous study based on synoptic sampling of 168 locations in this same catchment (median: 3.3 g C m^−2^ day^−1^; Rocher‐Ros et al., 2019). Similarly, the median gas transfer velocity (*K*_600_ standardized by depth) in that synoptic study was much higher (54.5 m/day) than the median gas transfer velocity in this study (7.4 m/day), suggesting that reaches selected for metabolism estimates do not represent the most important locations for CO_2_ evasion in the network. Indeed, if the median NEP from this study (1.4 g C m^−2^ day^−1^) is representative of the catchment, this would indicate that stream NEP only accounts for 40% of the CO_2_ evasion estimated from the more spatially extensive sampling effort. This contribution is more similar to other studies (Hotchkiss et al., 2015; Lupon et al., 2019; Rasilo et al., 2016). Thus, without a spatial assessment of CO_2_ evasion that included other hotspots of C inputs and evasion (Rocher‐Ros et al., 2019), the conclusion of this study would have overestimated the contribution of in‐stream metabolism. This stresses the importance of combining different tools, approaches and scales that capture unique pathways for C processing and evasion in stream networks.

While stream respiration appears to be important for CO_2_ evasion, capturing these rates and understanding their underlying drivers remain sources of uncertainty. Respiration rates in streams can be regulated by temperature (Demars et al., 2016; Song et al., 2018) and organic C supply: either autochthonous (i.e. GPP; Huryn et al., 2014) or allochthonous (i.e. litterfall or DOC; Demars, 2018; Roberts, Mulholland, & Hill, 2007). In our study, ER was strongly related to discharge (Figure S10), which has been reported elsewhere for other small northern streams (Demars, 2018; Lupon et al., 2019). These authors suggest that discharge could regulate the activity of stream heterotrophs through the delivery of terrestrial organic C. Consistent with this supply mechanism, previous work in the Miellajokka catchment has shown that DOC increases with discharge (Giesler et al., 2014). Thus, the positive relationship between ER and discharge reported here could reflect real hydrological processes that drive the supply and processing of terrestrial organic C in these streams.

Despite this plausible mechanism, the close correspondence between ER and discharge needs to be taken with caution because it also reflects covariance between *K*_600_ and ER (Figure S12). In this study, the observed relationship between ER and discharge (or *K*_600_) emerges from a persistent deficit of O_2_ across a large range of flow conditions (see Figures S2 and S3). The covariance between ER and another parameter such as *K*_600_ can be problematic when studying within site variability of ER, and so we are conservative and focus on average rates of ER, as other studies have done (Blaszczak, Delesantro, Urban, Doyle, & Bernhardt, 2018). Regardless, since *K*_600_ is used both for metabolism modelling and for CO_2_ evasion (Equations 4 and 5), potential biases arising from *K*_600_ estimates would affect NEP and CO_2_ evasion rates to a similar extent and direction. This is also reflected in the similar departure from equilibrium for both O_2_ and CO_2_ (Figure 5a), which indicates a strong coupling of both gases in these streams. Finally, mass balance estimates of CO_2_ production provided an independent validation of NEP rates, which were remarkably similar to NEP measured via metabolism modelling in the same stream on the same day (2.6 g C m^−2^ day^−1^ vs. 2.8 g C m^−2^ day^−1^; Figure 6). Together, these multiple observations provide additional confidence in our conclusions regarding the important role of aquatic respiration to CO_2_ evasion.

Strikingly, our results further suggest that rates of NEP can exceed evasion locally, leading to an accumulation and downstream export of CO_2_ (Figure 5). While this condition (NEP > E_CO2_) was evident from our continuous, modelled data, we also tested whether this is reasonable using a mass balance approach. In this case, along a 2 km stream reach, we observed a large increase of *p*CO_2_ (Figure 6). Given that this is a hydrologically loosing reach, most of the CO_2_ must be produced internally and thus originate from stream processes. Furthermore, continuous sensor data identified this reach as one of the sites where NEP > E_CO2_ (Site M1_LK_ in Figure 5). Together, these observations suggest that in‐stream biological processes can actively generate CO_2_ in streams and override lateral transport. Overall, whether a stream reach can or cannot export CO_2_ downstream will ultimately be controlled by the turbulence of the water and the capacity to evade CO_2_, which is highly variable at fine spatial scales (Rocher‐Ros et al., 2019). The interplay between stream reaches that are importers or exporters of CO_2_ creates a strong heterogeneity and dynamism within stream networks that has important implications for understanding how C is processed and evaded along the aquatic continuum.

### 4.3 Understanding the effects of climate change for C cycling in high‐latitude streams

The Arctic is currently confronted by a wide array of changes due to global warming, with increased temperatures that result in the mobilization of old OC in soils (Schuur et al., 2015) and increased discharge into the Arctic Ocean (Peterson et al., 2002). These changes are currently altering the functioning of stream ecosystems (e.g. Kendrick et al., 2018) and also appear to have strong effects on CO_2_ evasion from fluvial networks (Serikova et al., 2018). Our results suggest that Arctic streams and rivers not only play an important role in C export and CO_2_ evasion, but are active components in the biological mineralization of OC.

However, the Arctic is large and diverse, with variation in permafrost extent and soil C storage (Hugelius et al., 2014), as well as regional differences in vegetation structure and growth trends (Huang et al., 2017), which together underpin largely unknown variability in stream biogeochemistry and aquatic ecosystem dynamics. Thus, multiple Arctic regions may respond uniquely to global change, and this variability needs to be captured in future studies, given current focus on few Arctic areas (Metcalfe et al., 2018). Regardless, owing to the importance of the Arctic C feedback on climate change (Schuur et al., 2015) and the dependence of stream respiration to discharge and C supply (Demars, 2018), we suggest to include stream metabolism and its response to environmental change (e.g. Song et al., 2018) in future scenarios for the prediction of the effects of climate change.

## CONFLICT OF INTEREST

The authors declare no competing interests.
