# Supplementary material for: Stream metabolism controls diel patterns and evasion of CO2 in Arctic streams
Source: Glob Chang Biol. 2019 Nov 29;26(3):1400–13. doi: 10.1111/gcb.14895 (PMC7078971; doi:10.1111/gcb.14895)
Supplement: Supplementary file 1 [file GCB-26-1400-s001.docx]

Supplementary Materials:

**Stream metabolism controls diel patterns**

**and evasion of CO_2_ in Arctic streams**

**Authors**

Gerard Rocher-Ros^1*^, Ryan Sponseller^1^, Ann-Kristin Bergström^1^, Maria Myrstener^1^, Reiner Giesler^1^

**Affiliation**

^1^Climate Impacts Research Centre, Department of Ecology and Environmental Science, Umeå University, 98107 Abisko, Sweden

**Correspondence:** Gerard Rocher-Ros ([g.rocher.ros@](mailto:g.rocher.ros@)gmail.com)

**Table of Contents:**

**Figure S1**: Time series of CO_2_, O_2_ and discharge 2

**Figure S2:** Relationships between discharge and O_2_ 3

**Figure S3:** Conceptual responses of ER and O_2_ saturation with discharge 3

**Figure S4**: Relationships between discharge and K_600_ 4

**Figure S5**: Stream metabolic rates 5

**Figure S6**: Metabolic rates in a global context 6

**Figure S7:** Comparison of prior and posterior K_600_ 6

**Figure S8**: Relationship between GPP and diel change in CO_2_ concentration 7

**Figure S9**: Relationship between GPP and ER 7

**Figure S10:** Relationship between discharge and ER 8

**Figure S11**: Relationship between discharge and GPP 8

**Figure S12:** Relationship between K_600_ and ER 9

**Figure S13**: Map of the stream reach of the mass balance 10

**Table S1**: Coefficients of the linear regressions between NEP and E_CO2_ 11


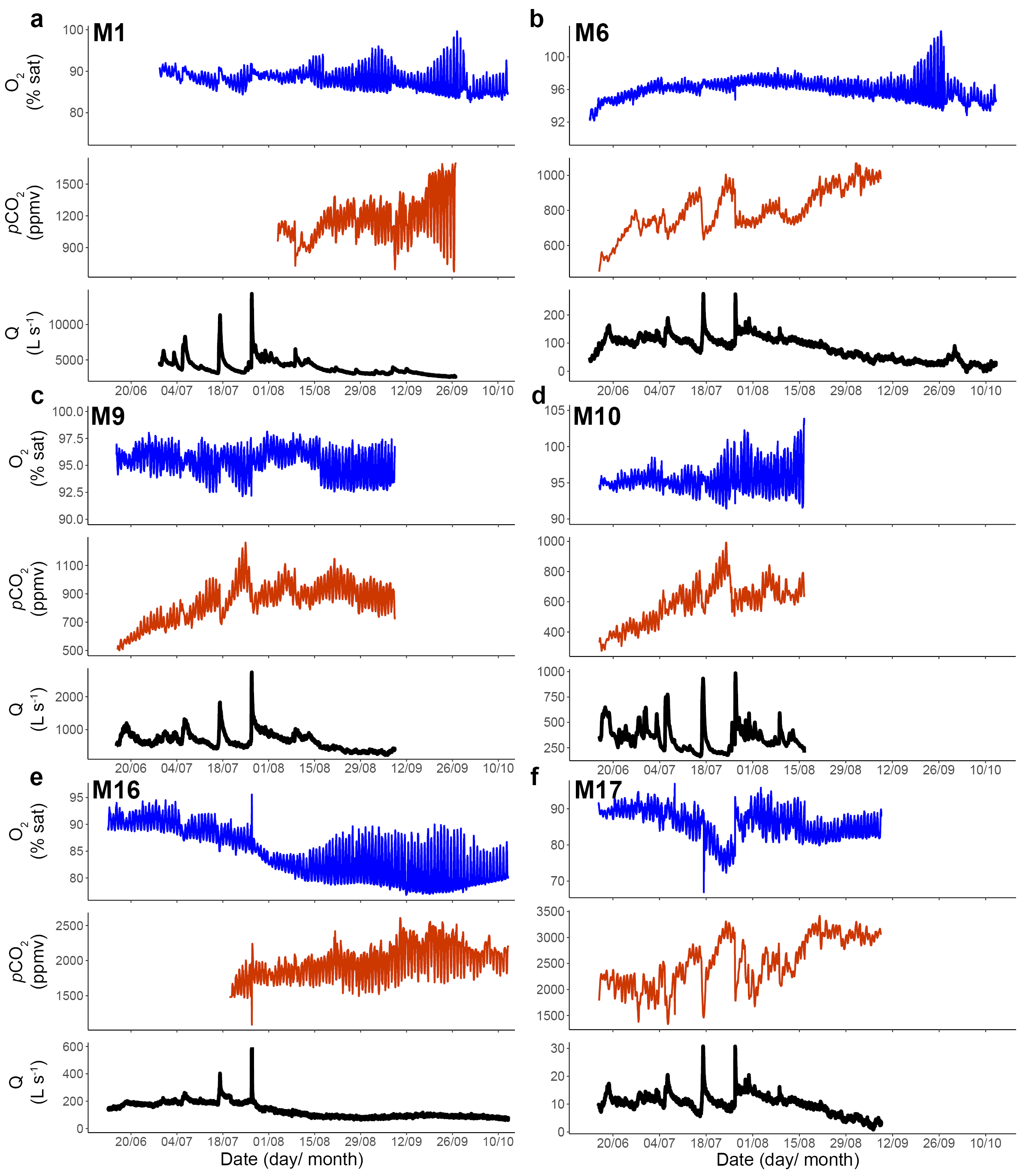


**Figure S1**. Time series of discharge, CO_2_ and O_2_ for the six streams monitored in 2016. Each panel (a), (b), (c), (d), (e) and (f) represent the streams M1, M6, M9, M10, M16 and M17 respectively. The upper plot shows the O_2_ saturation in % (blue), the mid plot shows the *p*CO_2_ (red), and the lower plot is discharge in L s^-1^ (black). The x-axis is consistent across all plots but the y-axis changes due to large differences of the parameters across sites.


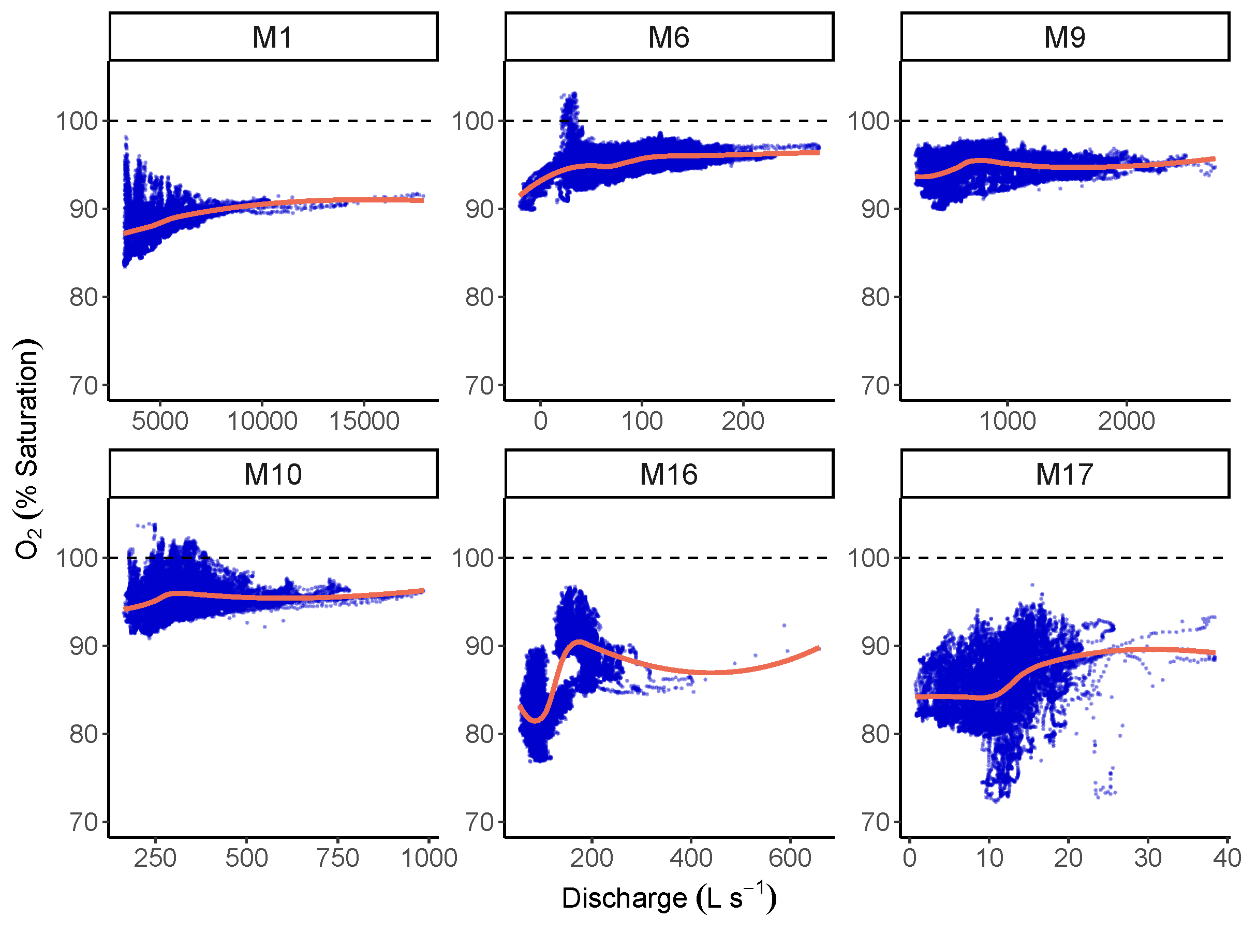


**Figure S2**. Relationship between discharge and O_2_ concentration (expressed as relative saturation) for the six sites in the study. Each point is an observation at a 10-minute resolution. The orange line is a “loess” fit, representing a moving average of the data, while the dashed line is the 100% saturation and equilibrium with the atmosphere.


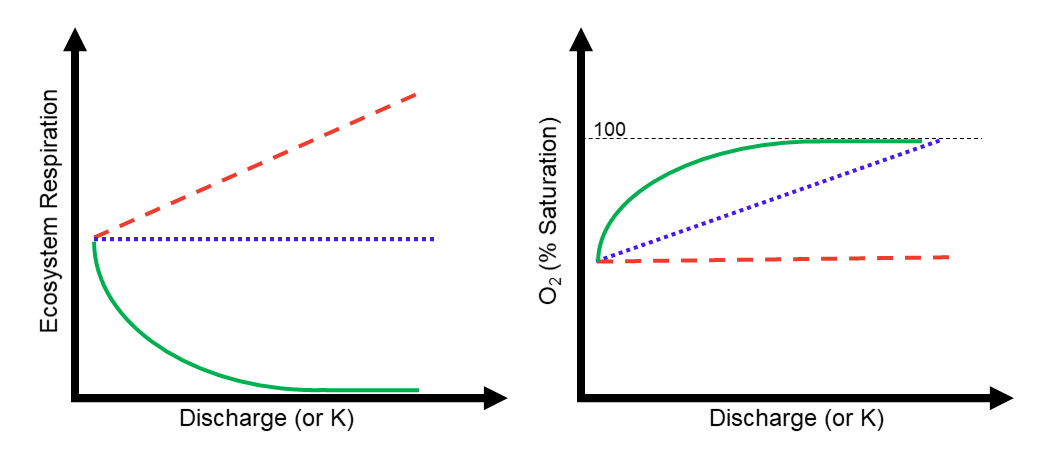


**Figure S3**. Potential responses of ecosystem respiration (ER) with discharge (Q), depending on changes in oxygen saturation (b). In this study K is linearly related with discharge (figure S4). Thus, for ER to respond negatively with Q, the O_2_ saturation should rapidly approach equilibrium with the atmosphere (green solid line). For ER to remain constant (blue dotted line), the oxygen saturation should also increase, to maintain a similar influx of oxygen due to the increased K. If ER increases with Q, the oxygen saturation should remain relatively constant and not reach equilibrium with the atmosphere (red line). In this study, our observations indicate that oxygen saturation remains relatively constant despite increases in discharge and K_600_ (Figure S2). Thus, the influx of oxygen (due to ER) needs to increase with discharge to maintain a given deficit of oxygen in the water (Figure S10).


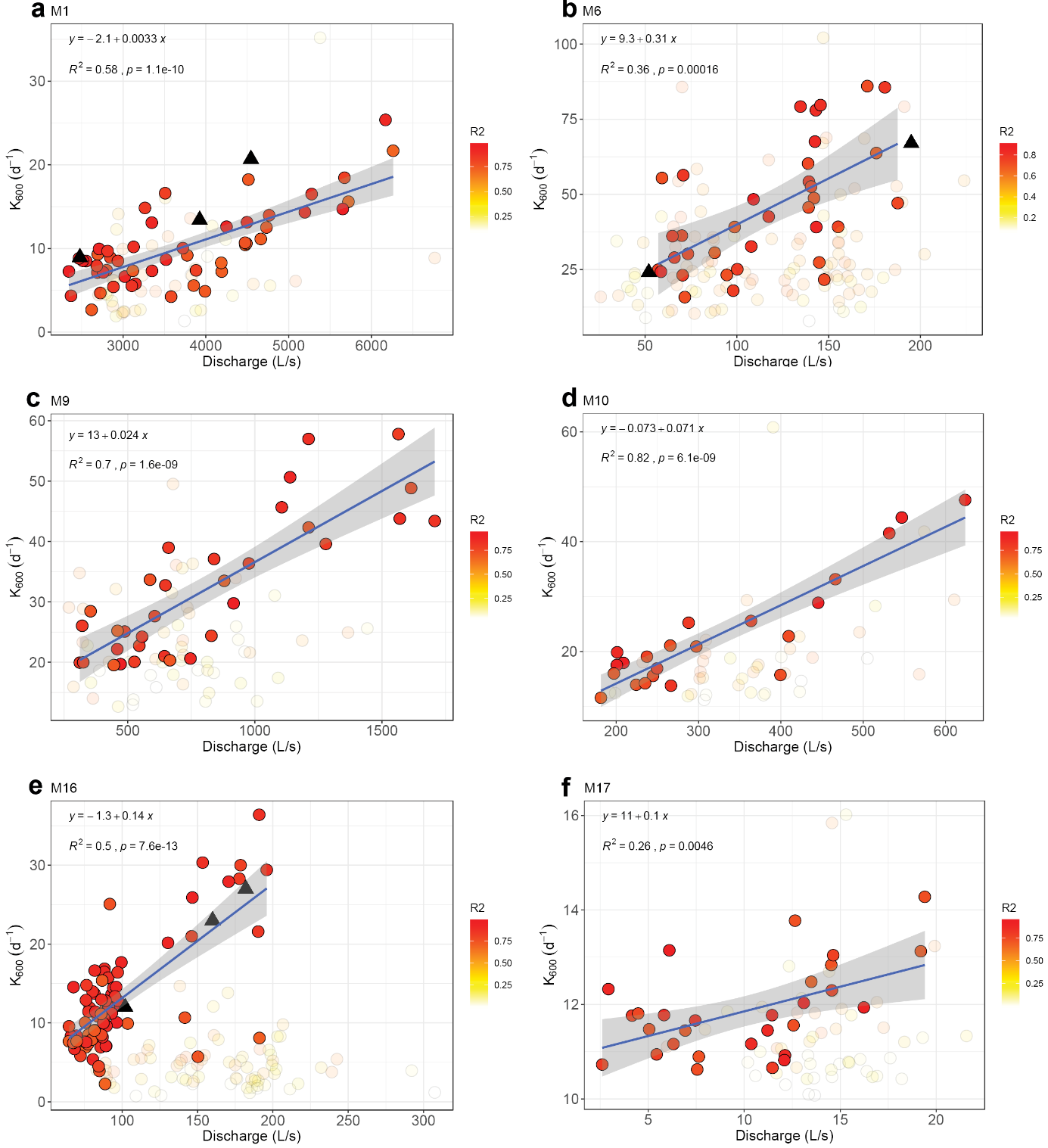


**Figure S4**. Relationship between discharge and K_600_ for the six streams. Each plot (a), (b), (c), (d), (e) and (f) represent the streams M1, M6, M9, M10, M16 and M17, respectively. The K_600_ were obtained from the night-time regression method. The colour of the points represents the R^2^ value of the nigh-time regression for each day. The black triangles show K_600_ values obtained from propane injections, wherever that was feasible. For the Q~K_600_ model we used days when the night-time regression had an R^2^ ≥ 0.7. The days when R^2^ < 0.7 are shown with high transparency in the plot. At the site M1, the triangles correspond to propane K measurements from another study, performed 500m upstream and in 2014 (Erik Lundin, personal communication).


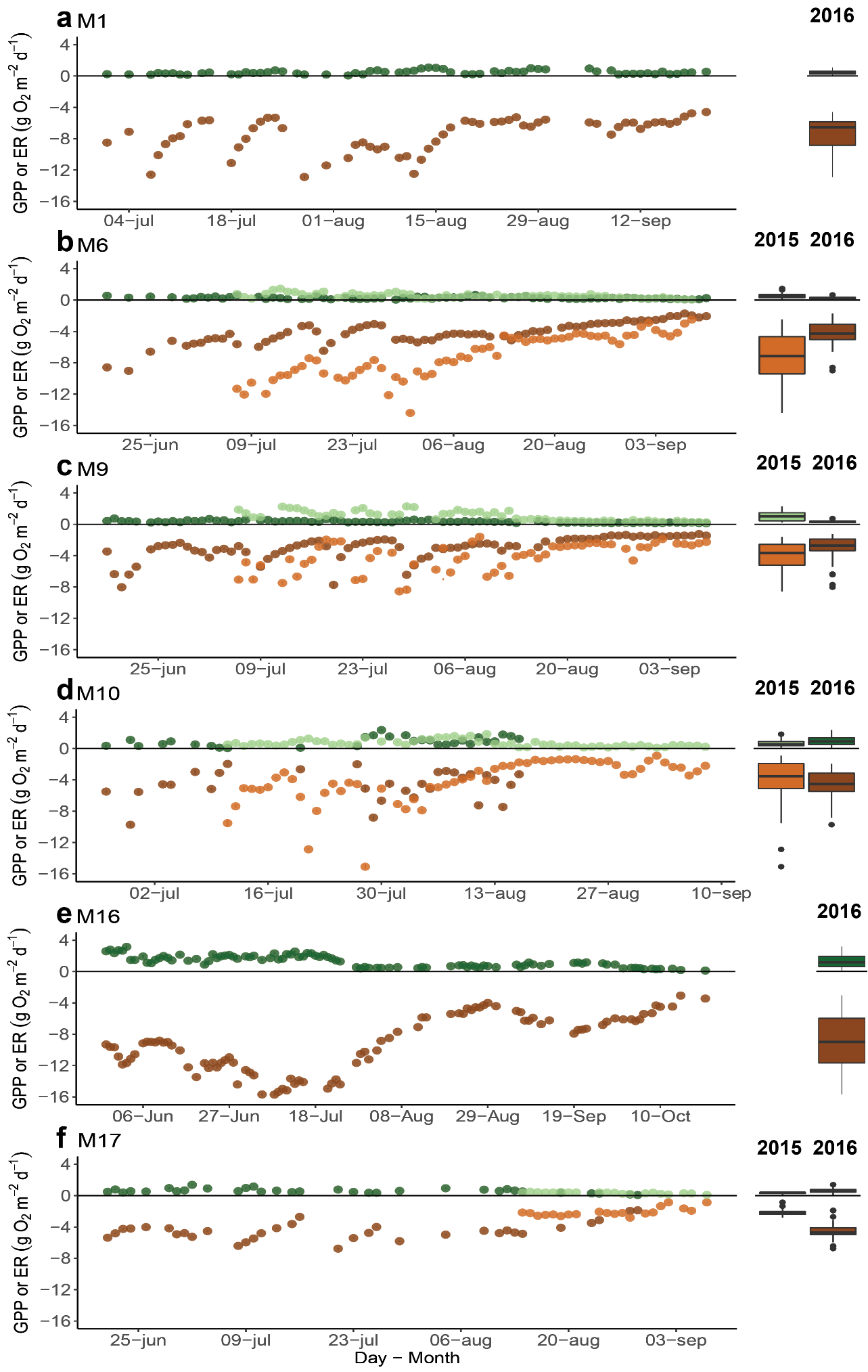


**Figure S5.** Metabolism time series of the six sites in the Miellajokka catchment. Gross primary production (GPP) are shown in green and ecosystem respiration (ER) in brown, while light colours are for 2015 and dark colours for 2016 data points. Owing to the size of symbols, the 0.05-0.95 credible intervals are not visible in the plot.


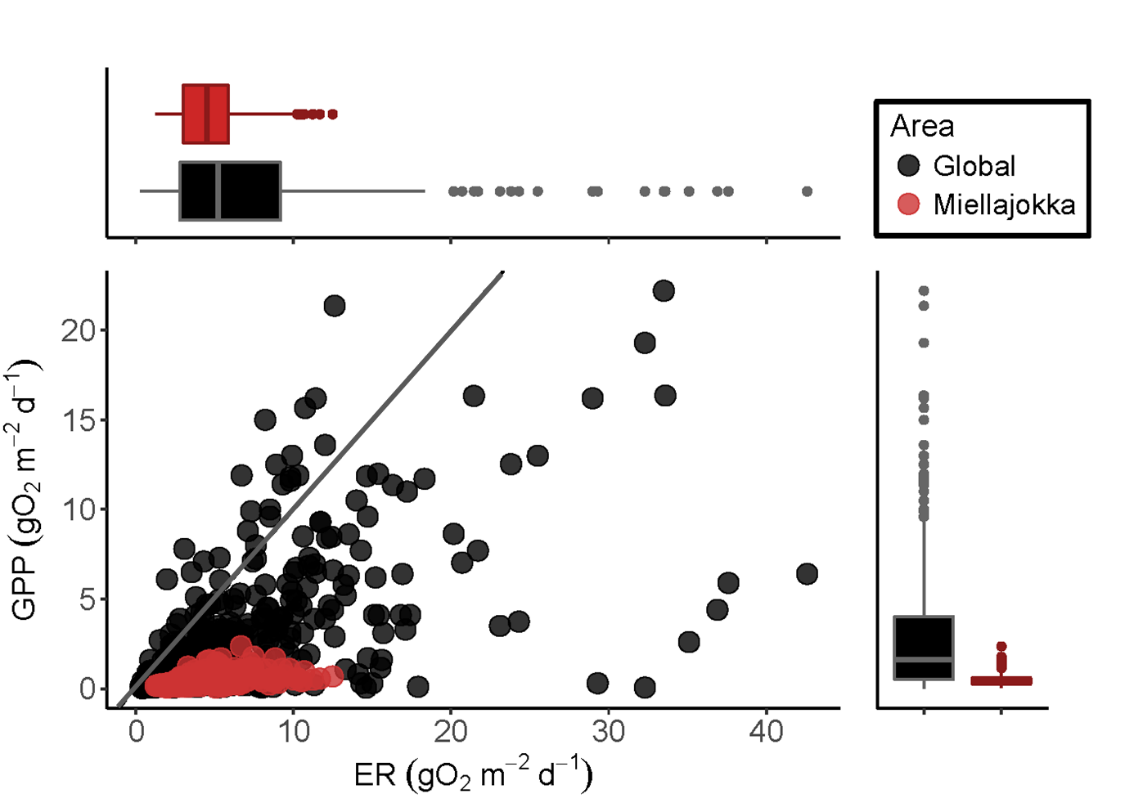


**Figure S6.** Metabolic rates in this study compared to a global compilation (Hoellein et al., 2014). The grey line denotes the 1:1 line and thus points on the top-left corner are autotrophic and on the bottom-right heterotrophic. The boxplots on top and the side represent the distribution of ER and GPP respectively, with the box indicating the interquartile range (IQR), the inner bar the mean, and the points are values higher or lower than 1.5*IQR.


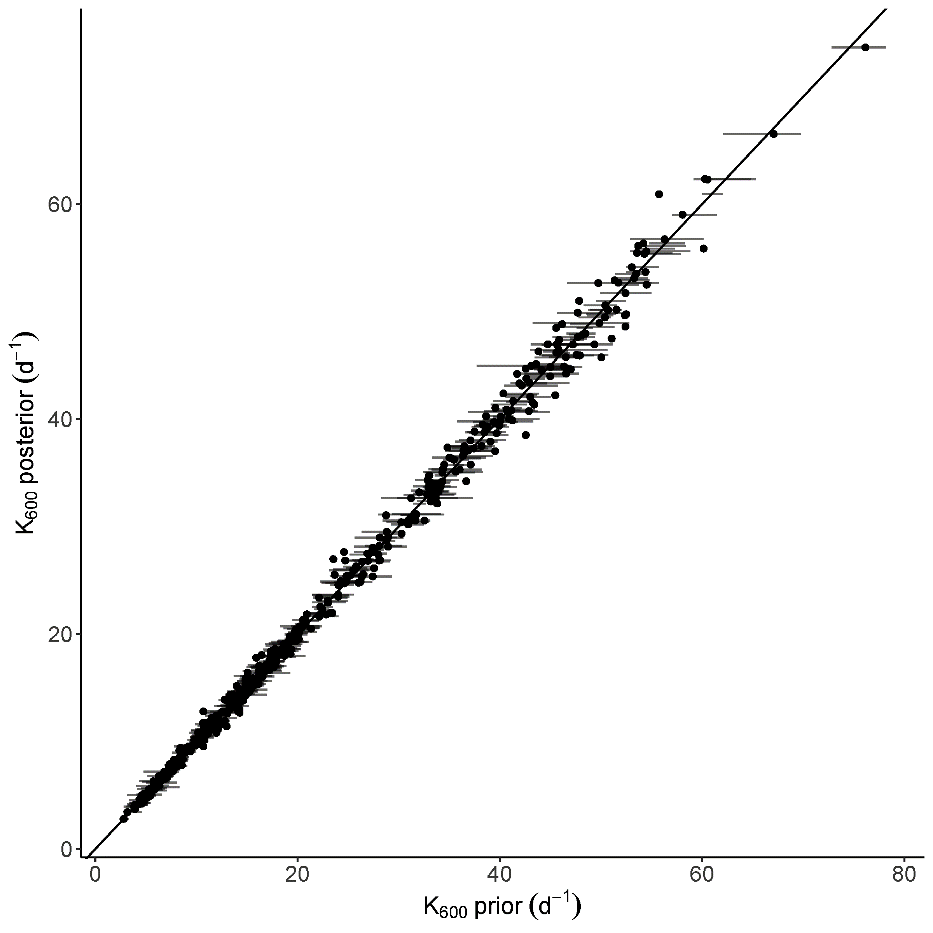


**Figure S7.** Relationship between the prior K supplied to the metabolism model and the posterior K chosen by the model. The segment for each point represents the 0.05-0.95 credible interval.


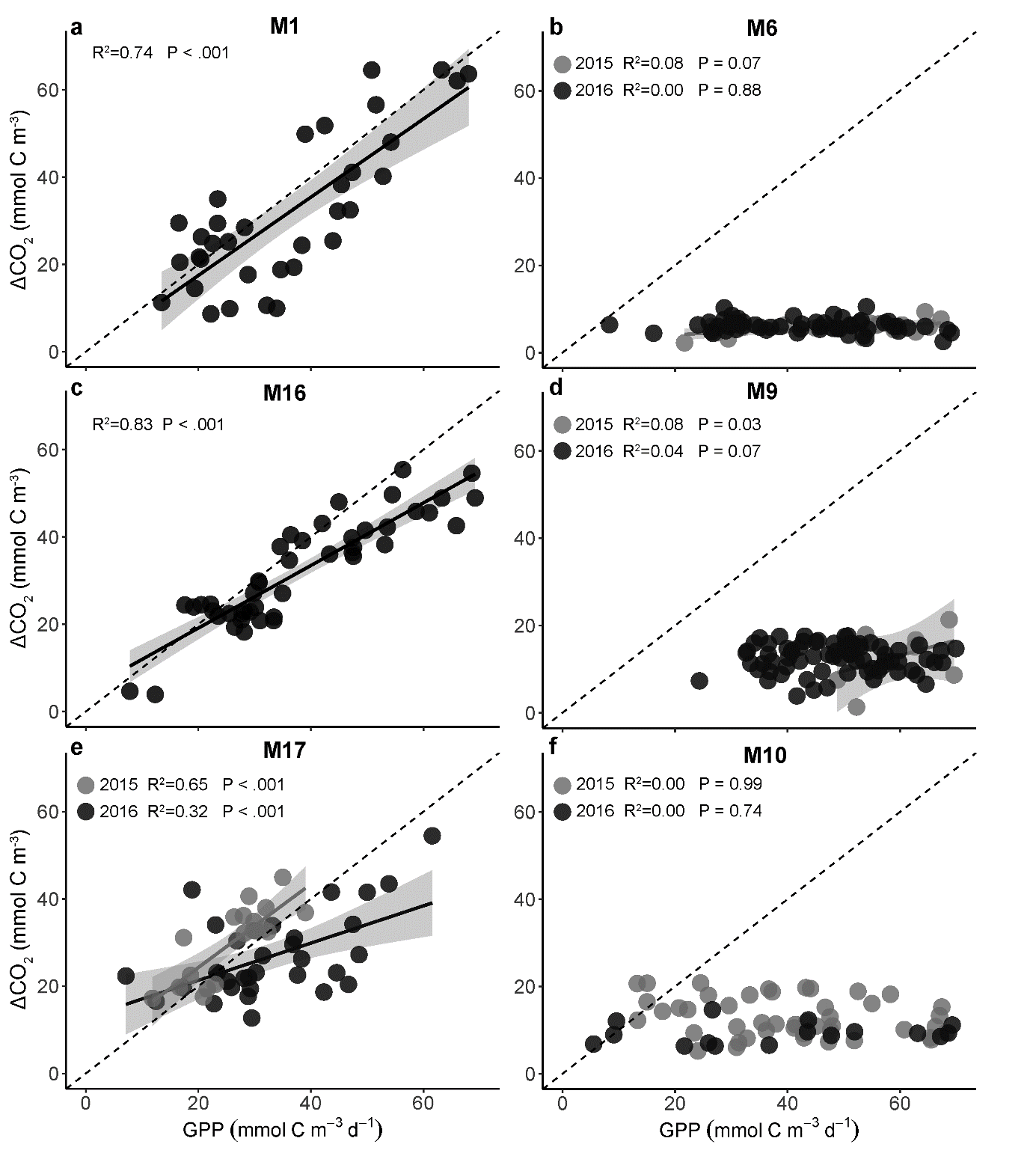


**Figure S8**. Relationship between GPP and the diel change in *p*CO_2_ for all streams. This is a similar plot as in figure 3 and 4, but showing observed changes in *p*CO_2_.


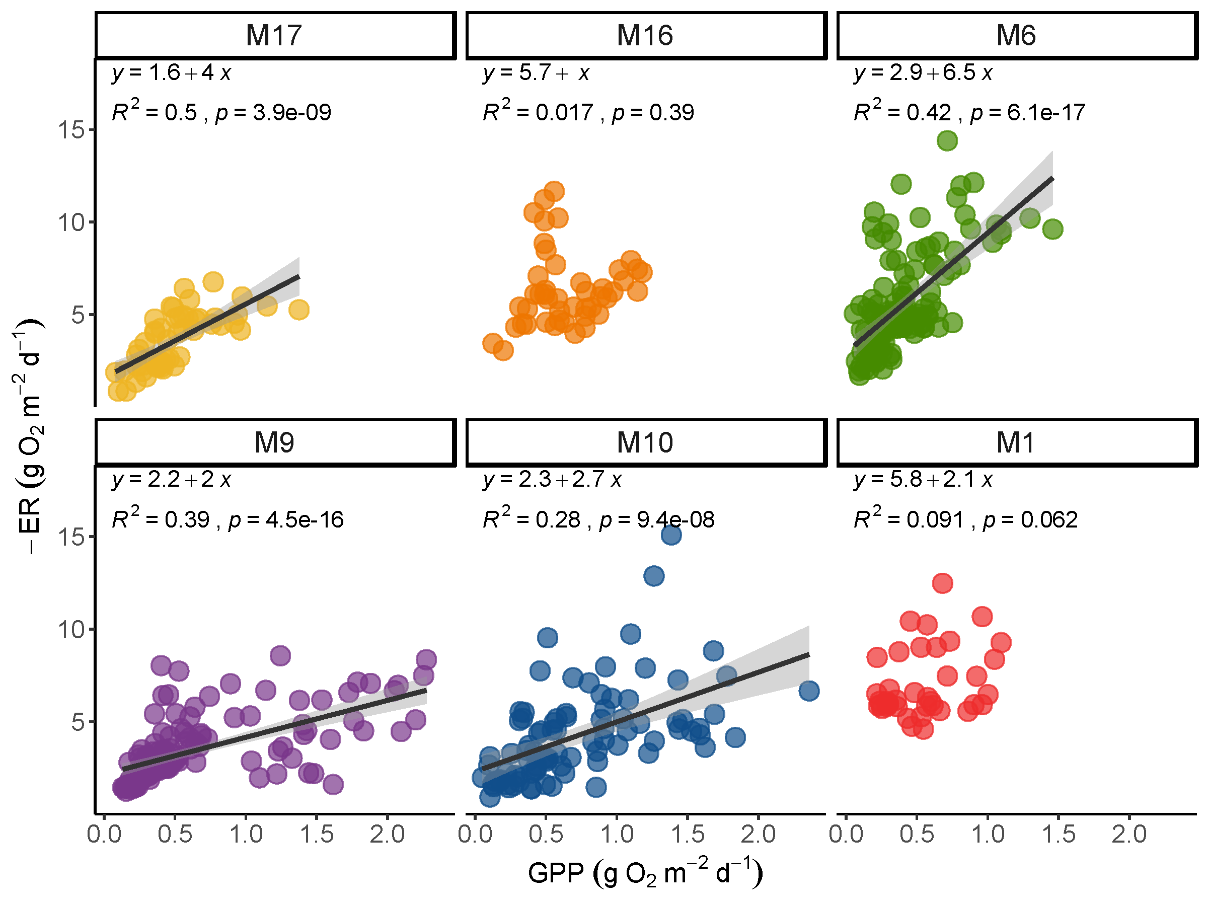


**Figure S9.** Relationship between gross primary production (GPP) and ecosystem respiration (ER) for each site. There was a significant relationship between ER and GPP for all sites except M1 and M16 (p-value>0.05).


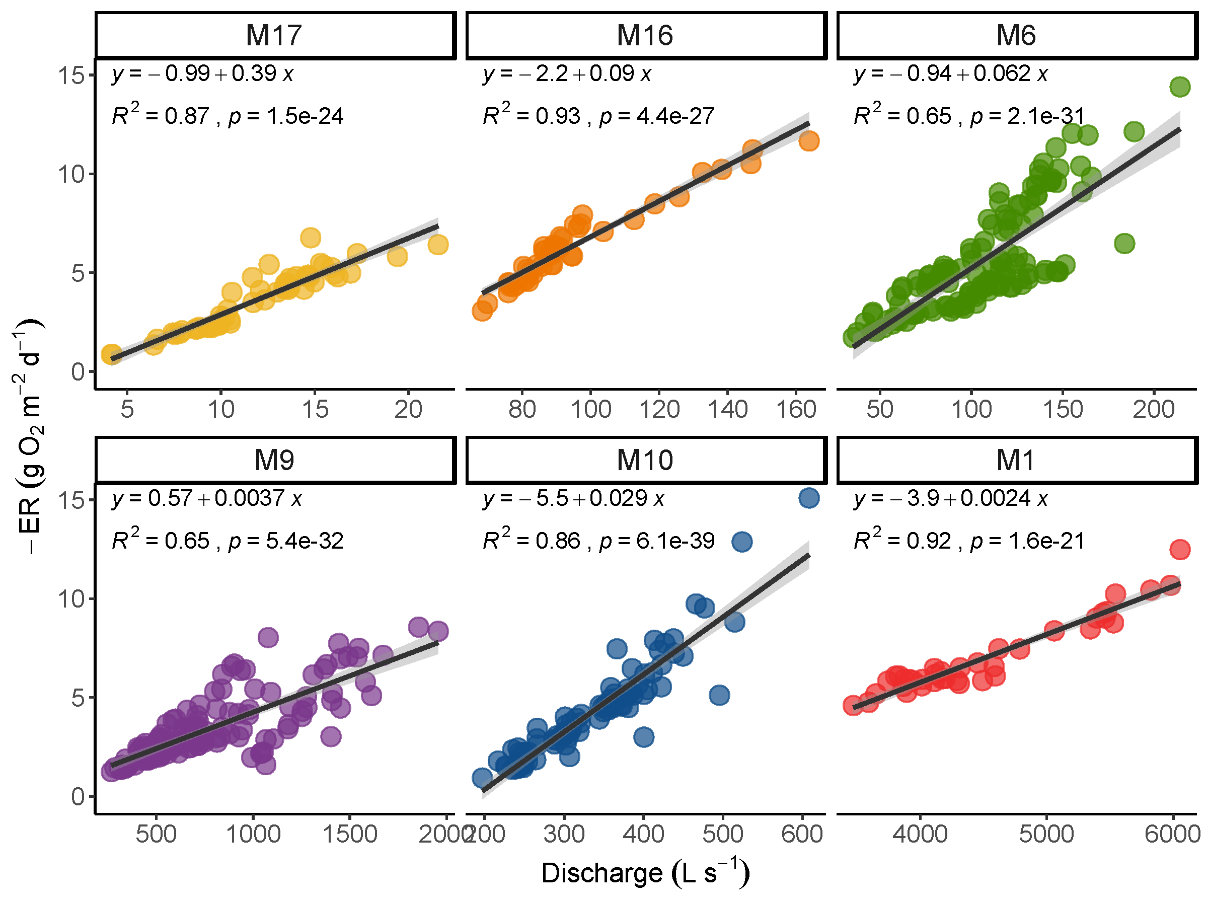


**Figure S10.** Relationship between discharge and ecosystem respiration (ER) for each site. As K_600_ was linearly modelled from discharge (Figure S2), ER and K_600_ are also linearly related with a similar strength (Figure S8).


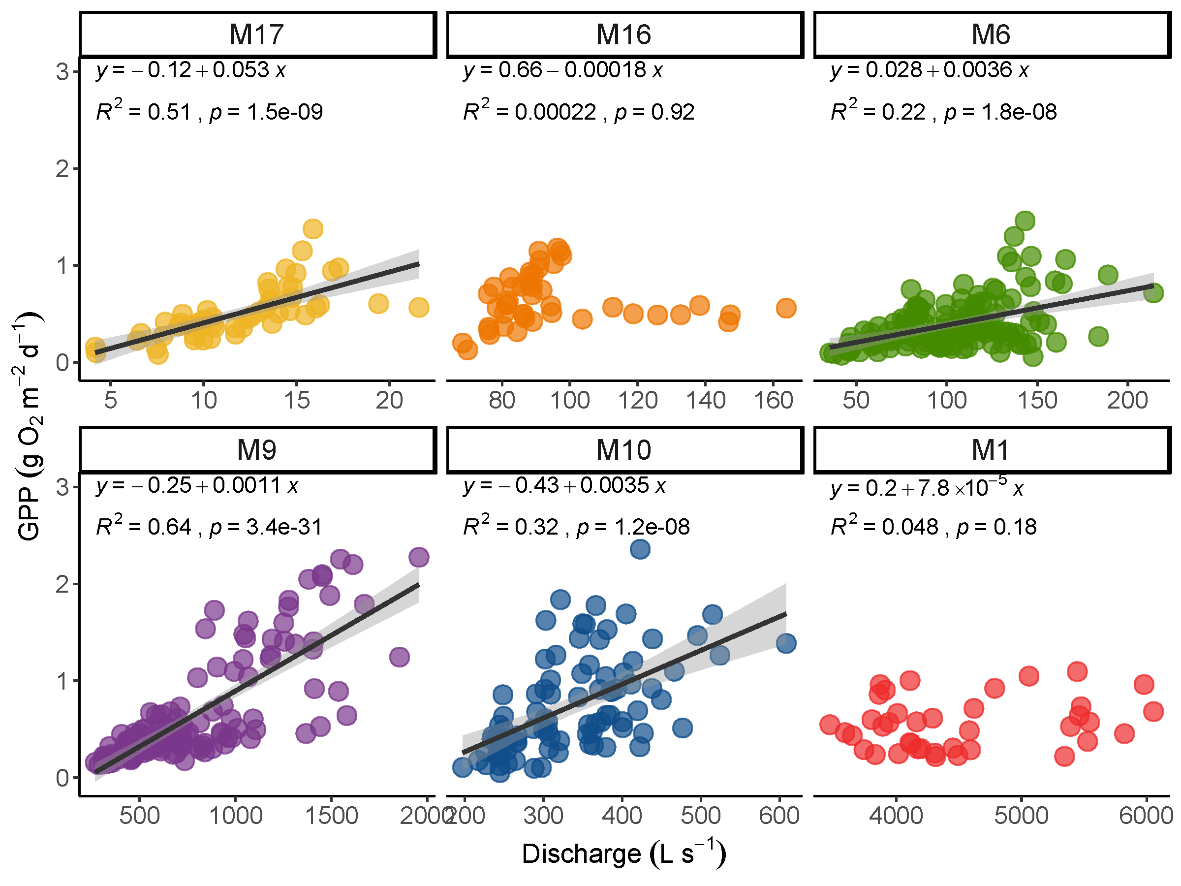


**Figure S11.** Relationship between discharge and gross primary production (GPP) for each site.


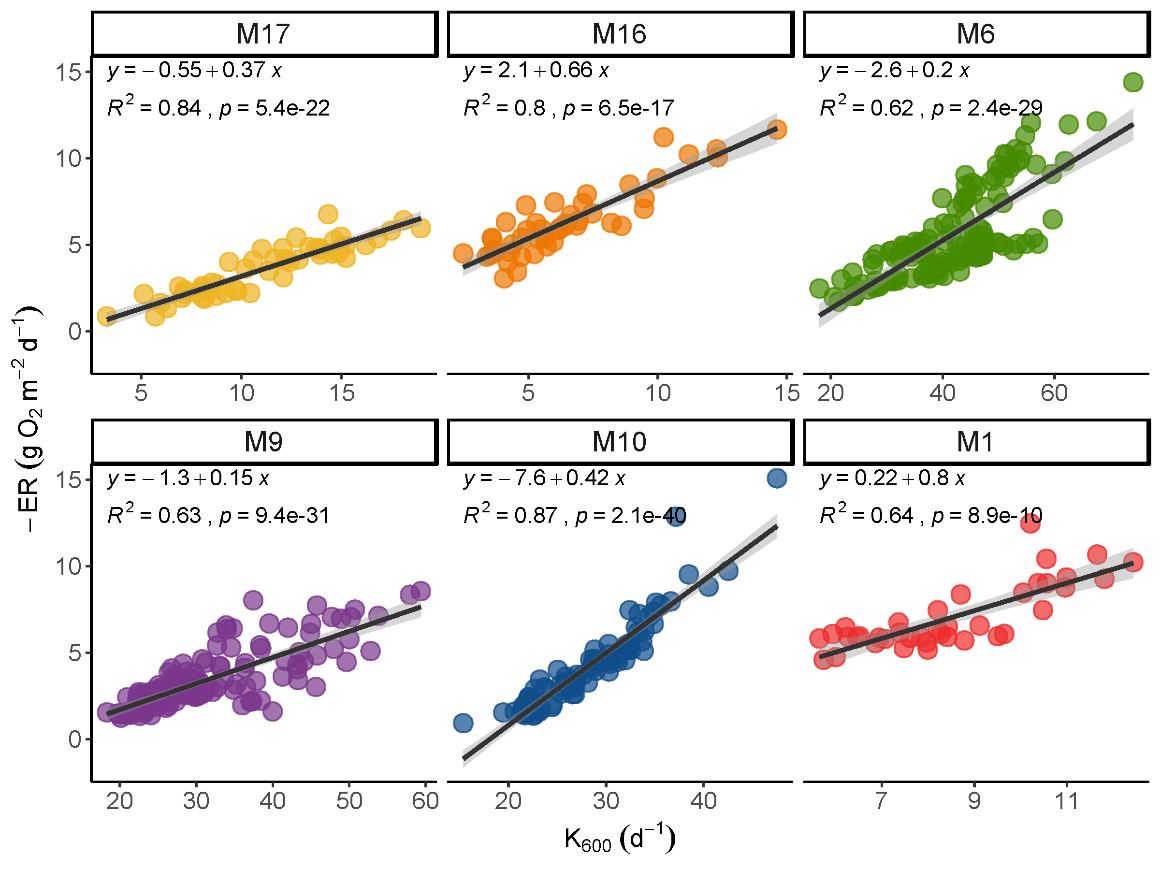


**Figure S12.** Relationship between K_600_ and ecosystem respiration (ER); K_600_ and ER were significantly linearly related in all stream sites.


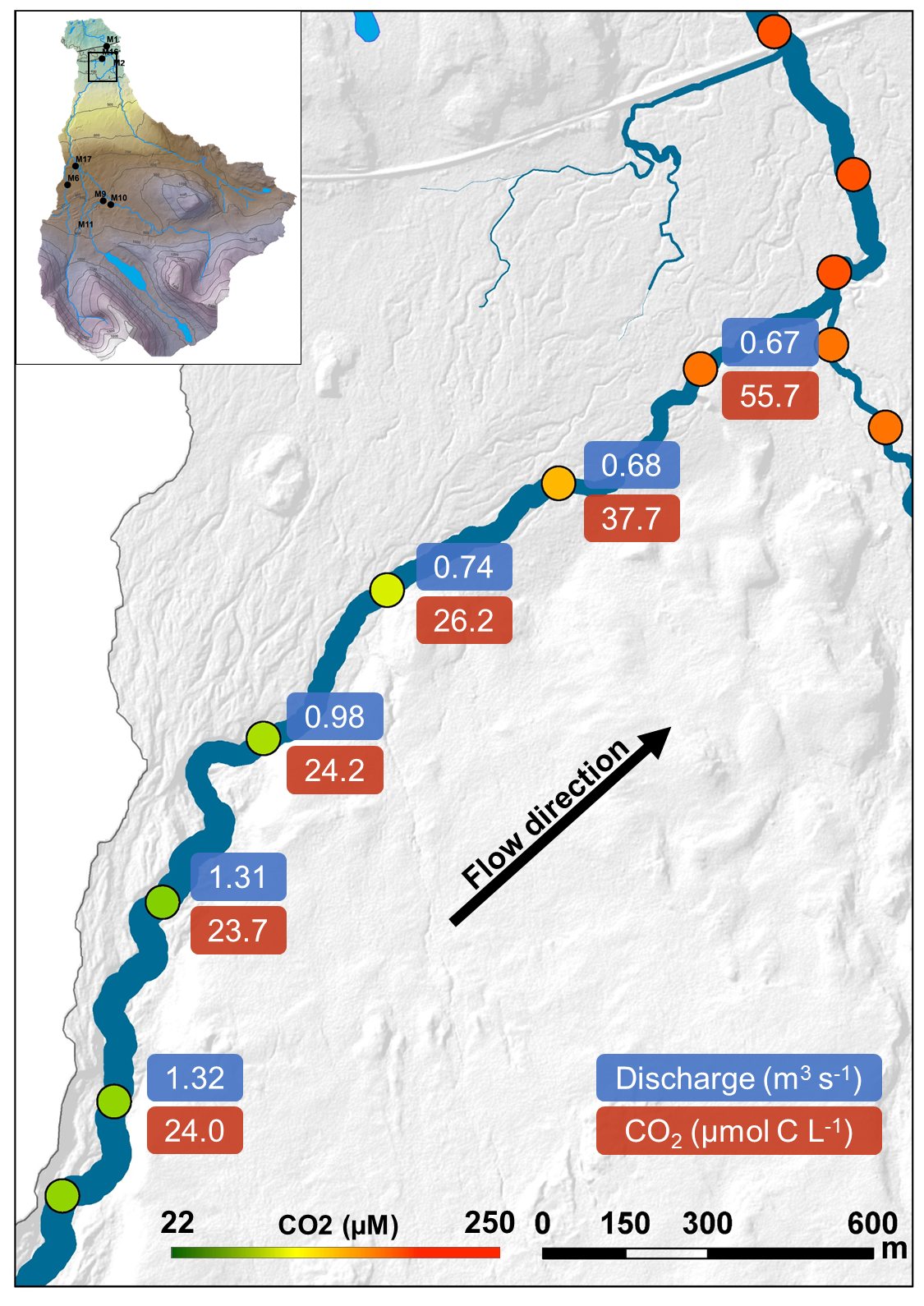


**Figure S13:** Spatial measurements of CO_2_ and geomorphological properties, using the dataset from Rocher-Ros et al., (2019), measured the 20^th^ of July of 2016. The map shows a LIDAR image with the stream network at the northern end of the catchment (see inset for the location). The line width of the stream is proportional to discharge, and the colour of the points represents the CO_2_ concentration at each measuring point. From the LIDAR image, paleo-stream channels are visible that feed the alluvium deposits (left side of the stream reach). At the end of the alluvium, a small headwater system emerges and drains into the main stem.

**Table S1**: Coefficients of the linear regression CO_2_ evasion ~ NEP, in figure 5b.

| **Site** | **Intercept** | **Slope** | **Adj. R^2^** | **p-value** |
| --- | --- | --- | --- | --- |
| M1 | 0.40 | 0.38 | 0.74 | < 0.001 |
| M6 | 1.06 | 0.27 | 0.10 | 0.005 |
| M9 | 0.38 | 0.56 | 0.23 | < 0.001 |
| M10 | 0.56 | 0.10 | 0.00 | 0.5 |
| M16 | -0.36 | 1.41 | 0.96 | < 0.001 |
| M17 | 0.50 | 0.88 | 0.71 | < 0.001 |
